# Linking speech patterns to brain structure in affective and psychotic disorders: an integrative natural language processing approach

**DOI:** 10.1038/s41380-025-03347-9

**Published:** 2025-11-20

**Authors:** Svenja Seuffert, Rieke Roxanne Mülfarth, Lea Teutenberg, Florian Thomas-Odenthal, Paula Usemann, Nina Alexander, Hamidreza Jamalabadi, Igor Nenadić, Benjamin Straube, Tim Hahn, Udo Dannlowski, Tilo Kircher, Frederike Stein

**Affiliations:** 1 Faculty of Medicine, Department of Psychiatry and Psychotherapy, Philipps-Universität Marburg, Marburg, Germany; 2 Center for Mind, Brain and Behavior, Philipps-Universität Marburg, Marburg, Germany; 3 Institute for Translational Psychiatry, University of Münster, Münster, Germany; 4 Bielefeld University, Medical School and University Medical Center OWL, Protestant Hospital of the Bethel Foundation, Department of Psychiatry, Bielefeld, Germany

**Keywords:** Neuroscience, Depression, Schizophrenia, Bipolar disorder

## Abstract

Language disturbances are central features of serious mental illnesses, yet traditional clinical assessments often rely on subjective evaluation that may overlook subtle speech anomalies. This study employs natural language processing (NLP) to objectively analyze spontaneous speech in a transdiagnostic sample comprising individuals with affective (*n* = 119 Major Depressive Disorder, *n* = 27 Bipolar Disorder) and psychotic disorders (*n* = 37 Schizoaffective Disorder, *n* = 11 Schizophrenia), as well as healthy controls (*n* = 178). Participants provided approximately 12 min of speech elicited via four pictures from the Thematic Apperception Test, which were transcribed and analyzed for semantic and syntactic features. Explorative factor analysis identified three latent linguistic dimensions: (1) *Syntactic Complexity,* (2) *Lexical Diversity and Fluency*, and (3) *Narrow Thematic Focus*. These dimensions were differentially associated with clinical ratings of formal thought disorder and neuroanatomical measures obtained through structural and diffusion-weighted MRI. Notably, *Syntactic Complexity* and *Lexical Diversity* correlated with decreased fractional anisotropy (FA) in frontotemporal white matter tracts, while *Narrow Thematic Focus* was linked to reduced gray matter volume in the right posterior insula. Importantly, these associations persisted after controlling for diagnosis, medication status, and verbal IQ. These findings suggest that NLP-derived speech metrics can serve as sensitive indicators for language dysfunction in psychiatric conditions, offering a scalable approach to elucidate brain-behavior relationships and advance models of psychopathology.

## Introduction

Language disturbances in mental disorders occur across diagnostic categories, including affective disorders such as Major Depressive Disorder (MDD) and Bipolar Disorder (BD), as well as psychotic disorders such as Schizophrenia (SZ) and Schizoaffective Disorder (SZA) [1–5]. These disturbances manifest as disorganized thought processes, incoherent speech, and difficulties in language production and comprehension, significantly impairing communication and social functioning [5–7]. Beyond clinical manifestations, language disturbances contribute to social isolation, diminished quality of life, and impaired functional outcomes. For instance, formal thought disorder (FTD) in SZ correlates with reduced psychological quality of life, underscoring the importance of addressing cognitive-linguistic deficits to enhance patient well-being [8]. Similarly, individuals with schizophrenia-spectrum disorders (SSD) or BD often experience impaired social functioning despite clinical remission, highlighting the need to identify mechanisms that hinder psychosocial recovery [9]. Moreover, the significance of FTD and speech and language abnormalities is underscored by their role in predicting disease onset in SSD and a more severe clinical course and a higher relapse risk in BD [5, 10] and MDD [11, 12], highlighting its prognostic and transdiagnostic relevance.

Multiple studies highlighted the transdiagnostic nature of FTD in general and speech and language abnormalities in particular [4, 13, 14]. Notably, Stein et al. [2] and Tang et al. [15] proposed three core transdiagnostic dimensions (i.e., *disorganization*, *emptiness*, *incoherence*) of FTD that were validated across MDD, BD, and SSD. Moreover, Stein et al. [2, 16] showed these transdiagnostic FTD dimensions to be correlated with gray matter volume (GMV) and whiter matter fractional anisotropy (FA) alterations as well as reduced whole brain structural connectivity. In addition, four different subtypes of FTD across affective and psychotic disorders were identified by applying a model-based cluster analytic approach [1]. The *minimal FTD*, *poverty*, *inhibition*, and *severe* FTD subtypes differed in general psychopathology, neurocognitive domain test performance as well as GMV and sulcal depth patterns. Interestingly, clinical diagnoses were distributed across subtypes and positive and negative FTD co-occurred [1].

Despite the utility of psychopathological FTD assessment scales, such as the Thought and Language Disorder scale (TALD) [13] and the Scale for the Assessment of Thought, Language, and Communication (TLC) [17], as well as general rating scales like the Scale for the Assessment of Positive Symptoms (SAPS) [18] and the Scale for the Assessment of Negative Symptoms (SANS) [19], these tools rely on clinical judgment and may lack sensitivity to subtle linguistic anomalies. Their subjectivity and limited scalability highlight the need for more objective approaches [20, 21]. Recent advances in Natural Language Processing (NLP), provide scalable, data-driven tools for quantifying language disturbances across lexical, syntactic, and semantic dimensions [22–27]. Using these approaches, researchers have identified patterns such as diminished semantic coherence, reduced syntactic complexity, and impaired discourse cohesion, which appear prominently in psychotic disorders [28–31] but also across affective disorders [32]. For example, reduced sentence complexity and fewer embedded clauses correlated with symptom severity in SSD [33]. In addition, Schneider et al. [34] demonstrated overlapping patterns of syntactic deficits in both SZ and MDD. Furthermore, impaired discourse cohesion and inappropriate conversational exchanges have been documented across psychiatric disorders. For example, individuals with SSD often use fewer contingency connectives (e.g., “because”), reflecting fragmented and less meaningful communication [35, 36]. Similarly, in MDD and BD, linguistic features like lexical diversity, affective language use, and negative emotional tone have been linked to symptom severity [37, 38]. By uncovering shared and disorder-specific features of language disturbances, computational approaches offer novel insights into the interplay between linguistic profiles and psychopathology.

Neurobiological investigations into language disturbances offer critical insights into their mechanistic underpinnings. In particular, FTD has been linked to alterations in both brain structure and function. Key brain regions in SSD included the frontal and temporal cortices, which are integral to language processing [4, 39, 40]. Disruptions in white matter tracts connecting these regions, including the uncinate fasciculus (UF), anterior thalamic radiation (ATR), and superior longitudinal fasciculus (SLF), are frequently implicated in SSD-related language dysfunctions assessed using clinical rating scales [41, 42]. In addition, the inferior fronto-occipital fasciculus (IFOF) has also been implicated in language-related abnormalities in psychosis. Surbeck et al. [43] reported that microstructural alterations of the left IFOF was associated with semantic processing deficits in SSD, while Maderthaner et al. [44] found that integrity was significantly related to dimensional measures of FTD in psychosis. In contrast, few studies investigated the association between NLP-derived metrices and brain structure and function, with the available evidence almost exclusively in SZ [45]. For example, Alonso-Sánchez et al. [46] found that higher semantic similarity in first-episode SZ patients’ speech was associated with increased inhibitory connectivity with the inferior frontal gyrus and ventral anterior temporal lobe. De Boer et al. [47] demonstrated that reduced lexical diversity and syntactic complexity correlated with decreased integrity in the SLF and IFOF. Additionally, Palaniyappan et al. [48] utilized automated speech graph metrics to relate disorganized speech to altered functional connectivity and cortical gyrification patterns.

Beyond SZ, recent transdiagnostic studies suggest that FTD-associated neural alterations span diagnostic boundaries, involving shared disruptions across affective and psychotic disorders [1, 2, 13, 16]. For example, Stein et al. [2] analyzed a large sample of patients with MDD, BD, and SSD, and demonstrated that the FTD dimension disorganization was negatively correlated with GMV in the left middle occipital and angular gyri, and positively correlated with FA in the right posterior cingulum bundle and the inferior longitudinal fascicle (ILF). In contrast, the emptiness dimension was associated with reduced GMV in the left hippocampus and thalamus, whereas incoherence was linked to diminished white matter integrity in the bilateral ATR and, conversely, to increased FA in the hippocampal segment of the right cingulum bundle. Building on these findings, Stein et al. [16] applied advanced network-based statistical analyses to further elucidate white matter connectivity patterns underlying FTD. They identified that both the disorganization and emptiness dimensions were associated with widespread white matter dysconnectivity. Importantly, these associations were robust across MDD, BD, and SSD, underscoring the transdiagnostic nature of FTD. Complementing these results, Schneider et al. [49] demonstrated that deficits in syntactic processing were associated across SZ and MDD patients with overlapping structural abnormalities in language-related white matter tracts, notably the SLF. Collectively, these converging lines of evidence support a dimensional framework for understanding FTD, suggesting that language disturbances in psychiatric disorders may reflect widespread alterations in both gray matter volume and white matter network connectivity, rather than being attributable to isolated regional deficits.

Despite these advances, investigations that cover several diagnostic categories remain scarce, particularly studies that integrate advanced NLP techniques with multimodal imaging modalities across diagnoses. This lack of integration has constrained progress in identifying shared neural mechanisms underlying language dysfunction across diagnosis. To address this gap, the present study combines NLP-derived linguistic dimensions with structural and diffusion-weighted imaging in a large, transdiagnostic sample. By mapping computational speech features onto brain structure, this multimodal design aims to enhance our understanding of the complex interplay between language and psychopathology and brain structure. To the best of our knowledge, this is the largest transdiagnostic study to date linking NLP-derived speech features to brain structure.

## Materials and methods

### Participants

Data were included from a sample of 372 individuals diagnosed with MDD (*n* = 119), BD (*n* = 27), or SSD (*n* = 48), as well as a control group of healthy individuals (HC, *n* = 178). Participants were from a subsample of the FOR2107 cohort [42], and diagnoses were confirmed using the Structured Clinical Interview for DSM-IV-TR Axis I Disorders (SCID-I) [50]. Inclusion criteria for patients were a primary diagnosis of MDD, BD, SZA, or SZ, aged between 18 and 69, and fluency in German. Healthy controls had no history or treatment of psychiatric disorders. Exclusion criteria for all participants included severe medical condition (e.g., neurological disorders, history of head trauma or unconsciousness), verbal IQ < 80, acute substance dependence, and current intake of benzodiazepines.

### Clinical assessment

Psychopathological assessment included a comprehensive battery of standardized clinical rating scales, as part of the semi-structured interview. These interviews were conducted by trained psychologists or researchers. Inter-rater reliability was assessed using intraclass correlation coefficients and showed good reliability across all scales (ICC = 0.86). Depressive and anxiety symptoms were evaluated using the Hamilton Depression Rating Scale (HAM-D) [51] and the Hamilton Anxiety Rating Scale (HAM-A) [52], while manic symptoms were assessed with the Young Mania Rating Scale (YMRS) [53]. Global functioning was quantified using the Global Assessment of Functioning (GAF) [50].

FTD-related symptoms were assessed using items from SANS [19] and SAPS [18]. The SANS measures negative FTD symptoms such as poverty of speech, poverty of content, blocking, and increased latency of response, while the SAPS focused on positive FTD symptoms, including derailment, tangentiality, incoherence, illogicality, circumstantiality, pressure of speech, distractibility, and clanging. Eight participants were excluded due to missing data on FTD items, resulting in a final sample of 364 individuals for FTD-related analyses. A full overview of clinical and demographic characteristics is provided in Table 1.

**Table 1:** Descriptive Statistics of the Speech Factor Analysis Sample (*N* = 372).

Variable	Healthy controls (*n* = 178)	Affective disorders (*n* = 146)	Psychotic disorders (*n* = 48)	p	Effect size
Age	44.36 (13.47)	44.32 (13.49)	41.17 (12.2)	0.306	
Sex, *n* (f/m)	114 / 64	91 / 55	18 / 30	0.003	V = 0.18
Years of education	14.56 (3.17)	13.42 (3.00)	12.02 (2.15)	**< 0.001**^**a,b,c**^	η² = 0.08
verbal IQ	116.06 (12.83)	115.56 (14.1)	111.89 (15.14)	0.180	
TIV	1530.03 (143.51)	1551.19 (153.63)	1565.62 (155.08)	0.294	
SANS nFTD subscale	0.25 (0.75)	1.15 (2.03)	2.70 (2.91)	**< 0.001 **^**d,e,f**^	η² = 0.18
SAPS pFTD subscale	0.53 (1.51)	1.82 (3.81)	5.83 (7.55)	**< 0.001 **^**d,e,f**^	η² = 0.17
SANS sum	1.33 (2.81)	7.79 (8.52)	18.13 (12.78)	**< 0.001 **^**d,e,f**^	η² = 0.37
SAPS sum	0.53 (1.28)	2.34 (4.97)	13.16 (13.8)	**< 0.001 **^**d,e,f**^	η² = 0.32
YMRS sum	0.40 (1.25)	1.44 (4.43)	6.00 (7.16)	**< 0.001 **^**d,e,f**^	η² = 0.18
HAM-D sum	1.33 (2.16)	5.66 (5.75)	9.24 (8.4)	**< 0.001 **^**d,e,f**^	η² = 0.25
HAMA-A sum	1.92 (2.80)	7.90 (7.33)	11.00 (10.06)	**< 0.001 **^**d,e,f**^	η² = 0.25
GAF score	85.36 (9.01)	65.94 (13.05)	51.91 (15.65)	**< 0.001**^**a,b,c**^	η² = 0.52

Mean (standard deviation), *TIV* total intracranial volume, *SANS* scale for the assessment of negative symptoms for the assessment of positive symptoms, *SAPS* scale for the assessment of positive symptoms, *nFTD* negative formal thought disorder, *pFTD* positive formal thought disorder, *YMRS* young mania rating scale, *HAM-D* hamilton rating scale for depression, *HAM-A* hamilton rating scale for anxiety, *GAF* global assessment of functioning, η² represents Eta-Squared, a measure of effect size in ANOVA that quantifies the proportion of variance in the dependent variable attributable to group differences; V represents Cramér’s V, a measure of effect size for Chi-square tests of independence that indicates the strength of association between categorical variables; significant results after Bonferroni correction for multiple testing are in bold.

^a^Affective disorders < Healthy controls.

^b^Psychotic disorders < Healthy controls.

^c^Psychotic disorders < Affective disorders.

^d^Affective disorders > Healthy controls.

^e^Psychotic disorders > Healthy controls.

^f^Psychotic disorders > Affective disorders

### Speech features

Participants were presented with four pictures from the Thematic Apperception Test (TAT) [54], a projective psychological instrument designed to elicit narratives that reveal underlying cognitive and emotional processes. Each participant was instructed to tell a story about what might be happening in the picture for three minutes per image, extending the typical one-minute storytelling duration used in earlier studies [55]. Speech was audio recorded and transcribed verbatim by trained linguists using the f4transkript software (https://www.audiotranskription.de/f4transkript), with transcribers blinded to diagnoses. NLP techniques were applied to extract a range of linguistic features across multiple domains. These included four lexical features (type-token ratio, measure of textual lexical diversity, pronoun and personal pronoun ratio), one morphological feature (morphological complexity), four syntactic features (syntactic complexity, subordination ratio, readability, and connective ratio), five semantic features (semantic coherence, sentence-level coherence, word-level coherence, semantic density, graph-based cohesion), three disfluency measures (filled pauses ratio, repetitions ratio, and grammatical errors), and one sentiment analysis (probability of negative sentiment). Feature extraction was performed using Python (version 3.11). The extracted features are summarized in Table 2. Details on how NLP features were extracted can be found in the Supplement (eMethod 1). Features were selected based on their relevance in psychiatric research and their potential to capture subtle language impairments associated with psychiatric conditions [15, 22, 33, 34].

**Table 2:** Description of Extracted NLP-Derived Speech Features.

Feature	Description
Type-Token Ratio (TTR)	Ratio of unique words (types) to total words (tokens), indicating vocabulary diversity.
Measure of Lexical Diversity (MTLD)	A measure of textual lexical diversity that accounts for word variation throughout the text.
Pronoun ratio	Proportion of pronouns (e.g., “he,” “they”) to total tokens, reflecting reference to entities.
Personal pronoun ratio	Proportion of personal pronouns (e.g., “I,” “we”) to total pronouns, indicating self-references.
Morphological complexity	Average number of morphological features per word, reflecting richness.
Syntactic complexity	Combined measure of average sentence length and depth of syntactic dependency trees, reflecting complexity.
Subordination ratio	Ratio of subordinate clauses to total clauses, indicating the use of complex syntactic structures.
Readability (Hohenheim Index)	A composite readability index combining sentence length and syllables per word, indicating text difficulty.
Semantic coherence (FastText)	Average cosine similarity between consecutive sentences based on word vectors, measuring coherence.
Sentence-level coherence (BERT)	Coherence based on BERT embeddings, assessing the semantic relationship between consecutive sentences.
Word-Level coherence (spaCy)	Semantic coherence based on spaCy word vectors across words in a document.
Semantic Density (content word density)	Average pairwise cosine similarity of content word vectors, indicating semantic richness.
Connective ratio	Proportion of connective words (e.g., conjunctions like “and,” “but”) to sentences, assessing logical structure.
Graph-Based Cohesion (Average Shortest Path Length)	Average shortest path length in word graphs, reflecting connectivity and cohesion in speech.
Filled Pauses	Proportion of filled pauses (e.g., “um,” “ah”) to total words, indicating hesitancy or interruptions.
Repetitions	Ratio of repeated words (consecutive identical words) to total words, reflecting stuttering or emphasis.
Grammatical error	Proportion of grammatical errors to total words, assessing deviations from standard language use.
Negative Sentiment	Probability of negative sentiment within the text, measured by a sentiment analysis model.

*NLP* Natural Language Processing.

### Neuroimaging acquisition and preprocessing

Structural magnetic resonance imaging (MRI) and diffusion tensor imaging (DTI) data were acquired using a 3 Tesla (3 T) MRI scanner (Tim Trio, Siemens, Erlangen, Germany) at the University of Marburg. MRI data acquisition followed an extensive quality assurance protocol to ensure data reliability and consistency [56]. After excluding participants due to missing data, poor image quality, motion artifacts, or incomplete scans, the final sample included 303 participants for GMV analyses and 247 participants for DTI analyses. Detailed description of the neuroimaging data acquisition and preprocessing are provided in the Supplement (eMethod 2). Descriptive statistics for these two neuroimaging samples are provided in the Supplement (Supplementary Table 1 and 2).

### Statistical analysis

Statistical analyses involving factor models and bivariate correlation analyses were conducted in R (version 4.4.1) [57]. An exploratory factor analysis (EFA) was conducted on the NLP speech features to identify underlying latent factors. The suitability of the data for factor analysis was assessed using the Kaiser-Meyer-Olkin (KMO) measure and Bartlett’s test of sphericity. Promax rotation was employed to allow for correlated factors, as the underlying factors were expected to be interrelated rather than orthogonal. The number of factors was determined using multiple empirical criteria. Robustness was assessed via bootstrapping, and an average factor solution was computed by integrating multiple extraction methods. A confirmatory factor analysis (CFA) was performed on the FTD items from SAPS and SANS to (re-)validate the factor structure identified in previous studies [2]. Full methodological details and criteria of the EFA and CFA are provided in the Supplement (eMethod 3). Correlation coefficients were calculated to examine the relationships between the NLP speech factors and FTD factors, applying Bonferroni corrections to adjust for multiple comparisons.

Voxel-based morphometry (VBM) analyses were conducted to explore the associations between NLP-derived speech factors and GMV. General linear models were employed for each NLP factor, incorporating age, sex, TIV, and diagnostic group as covariates. VBM analyses were performed using SPM12 and CAT12 with an absolute threshold masking value of 0.1. Results were considered significant at a cluster-level family-wise error (FWE) corrected threshold of *p*  <  0.05, following an initial uncorrected threshold of *p*  <  0.001 and a minimum cluster size of *k*  >  10 voxels. Cluster labeling was conducted using the Dartel space Neuromorphometrics atlas. For the DTI analyses, a tract-based spatial statistics (TBSS) approach was applied using the FMRIB Software Library (FSL) [58]. Threshold-free cluster enhancement (TFCE) and non-parametric permutation testing with 10,000 permutations were applied. Significance was set at *p*  <  0.05 FWE-corrected. Covariates for the TBSS analyses mirrored those used in the GMV analyses. Additionally, exploratory analyses were performed for radial diffusivity (RD), mean diffusivity (MD), and axial diffusivity (AD).

Moderation analyses were conducted to determine whether significant associations between NLP-derived speech factors and brain structures were influenced by verbal IQ and medication status. These moderators were chosen for their well-documented relevance in psychiatric research and speech. Verbal IQ, as a key measure of cognitive functioning, is linked to both linguistic abilities and brain structure, potentially confounding observed relationships [59, 60]. Medication status was included due to its known effects on brain morphology, such as changes in gray matter and white matter integrity [61, 62]. Therefore, eigenvariates of significant clusters were extracted in CAT12 and FSL.

## Results

### Speech data

The speech of the participants exhibited between 200 and 2496 words. On average, the speech exhibited 1139 words (*SD* = 370) and 86 sentences (*SD* = 29). The average length of a sentence per participants ranged between 6 and 36 words.

### Exploratory factor analysis of NLP speech features

An exploratory factor analysis (EFA) was applied to the NLP speech features to identify underlying latent NLP dimensions across diagnosis. The KMO measure indicated adequate sampling with a value of 0.82, and Bartlett’s test of sphericity was significant, χ²(136) = 1245.67, *p* < 0.001, confirming that the correlation matrix was suitable for factor analysis. Prior to conducting the EFA, we explicitly examined intercorrelations among all NLP-derived speech features and calculated variance inflation factors (VIFs). No pairwise correlations exceeded r = 0.80, and all VIF values were below 5, indicating the absence of problematic multicollinearity. A heatmap of the correlation matrix is provided in the Supplement (Supplementary Fig. 1). The EFA yielded a three-factor solution, explaining 65% of the total variance in the NLP speech features. To ensure the robustness of the factor structure, bootstrapping with *n* = 5000 was applied. Rotated bootstrapped loadings for the three-factor solution are presented in Table 3, with factors named based on the features demonstrating the highest loadings. Furthermore, an average three-factor solution was derived by integrating results from multiple EFAs performed using different extraction methods. The resulting average solution validated the stability and reliability of the factor structure across methodologies (see Supplementary Fig. 2). A distribution of speech factor scores across groups can be found in the Supplement (Supplementary Fig. 3).

**Table 3:** Bootstrapped Factor Loadings of Explorative NLP-Derived Speech Factors.

Speech Feature	Factor 1	Factor 2	Factor 3
Subordination ratio	**0.805**	0.140	0.242
Connective ratio	**0.801**	−0.108	0.203
Readability	**−0.796**	−0.251	0.308
Syntactic complexity	**0.743**	−0.167	−0.061
Semantic coherence	**0.695**	−0.295	−0.043
Graph-Based Cohesion	−0.215	**0.863**	0.237
Filled Pauses	0.103	**−0.815**	**−0.402**
Morphological complexity	−0.188	**0.544**	−0.200
TTR	−0.265	**0.511**	−0.223
MTLD	0.036	**0.511**	−0.317
Grammatical error	−0.290	**−0.435**	−0.330
Pronoun ratio	−0.267	0.196	**0.769**
Semantic Density	−0.121	−0.365	**0.573**
Word-level coherence	0.044	0.143	**0.570**
Sentence-level coherence	0.233	−0.111	**0.457**
Negative sentiment	−0.347	−0.088	0.144
Personal pronoun ratio	0.115	0.227	0.043
Repetitions	−0.114	−0.221	0.062

*N* = 372. The extraction method was unweighted least squares with an oblique (promax) rotation. Factor loadings above 0.40 are in bold.

*TTR* type-token ratio, *MTLD* measure of textual lexical diversity, Graph-Based Cohesion = average shortest path length in a speech graph. Factor 1: Syntactic Complexity; Factor 2: Lexical Diversity and Fluency; Factor 3: Narrow Thematic Focus.

#### Factor 1: syntactic complexity

Factor 1 was characterized by high positive loadings on features that represented syntactic complexity and cohesive discourse. Syntactic complexity (λ₁=0.743) and subordination ratio (λ₁=0.805) were significant contributors to this factor. Syntactic complexity measured the average depth of syntactic parse trees, indicating the complexity of sentence structures, while the subordination ratio reflected the proportion of subordinate clauses to total clauses, highlighting the use of complex grammatical constructions. The connective ratio also loaded highly on this factor (λ₁=0.801), indicating frequent use of connectives such as conjunctions and discourse markers that linked clauses and sentences, enhancing cohesion in speech. Semantic coherence, as measured by the FastText Coherence feature (λ₁=0.695), further underscored the logical and meaningful connections between concepts in discourse. Conversely, the Readability Index showed a strong negative loading (λ₁=−0.796) on this factor. Since the Readability Index is a metric where lower scores indicate more complex text, the negative loading aligned with the interpretation of this factor representing higher syntactic complexity.

#### Factor 2: lexical diversity and fluency

Factor 2 was defined by high positive loadings on features indicative of lexical diversity and speech fluency. The Graph-based cohesion loaded highest on this factor (λ_2_ = 0.863), suggesting that a more complex network of word associations contributed significantly to this dimension. Morphological complexity (λ_2_ = 0.544), Type-Token Ratio (TTR; λ_2_ = 0.511), and Measure of Textual Lexical Diversity (MTLD; λ_2_ = 0.511) all loaded positively, emphasizing the richness of vocabulary and morphological variation in speech. The ratio of filled pauses showed a strong negative loading (λ_2_ = −0.815), indicating that higher scores on this factor were associated with fewer disfluencies such as “um” or “ah.” The ratio of grammatical errors also negatively loaded (λ_2_ = −0.435), suggesting that grammatically accurate speech contributes to this factor.

#### Factor 3: narrow thematic focus

Factor 3 was defined by high positive loadings on pronoun ratio (λ_3_ = 0.769), semantic density (λ_3_ = 0.573), word-level coherence (λ_3_ = 0.570), and sentence-level coherence (λ_3_ = 0.457). Pronoun ratio measured the proportion of pronouns to total tokens, reflecting a tendency to use more general references rather than specific nouns, which simplified language. Semantic density calculated the average pairwise cosine similarity of content word vectors, indicating higher similarity between words—a characteristic of using common or less varied vocabulary. The positive loadings on word-level and sentence-level coherence suggested that despite simplification, the speech maintained coherence, possibly due to the use of straightforward language structures. These features suggested that Factor 3 captured aspects of narrow or focused narrative style, characterized by the frequent use of pronouns and semantically related content.

### Relationship between NLP speech factors and FTD factors

To explore the relationships between linguistic features and FTD symptoms, bivariate correlations were computed between the NLP speech factors and the FTD factors derived from clinical assessments (see eResults 2 in the Supplement for details on the CFA of FTD items). Results were corrected for multiple comparisons (Bonferroni corrected *p*-values). Factor 1 (Syntactic Complexity), correlated negatively with FTD Disorganization (*r* = −0.23, *p* = 0.002), FTD Emptiness (*r* = −0.21, *p* = 0.003), and FTD Incoherence (*r* = −0.18, *p* = 0.014). Factor 2 (Lexical Diversity and Fluency), correlated negatively with FTD Emptiness (*r* = −0.18, *p* = 0.016). Factor 3 (Narrow Thematic Focus) showed no significant correlations with FTD factors. Distributions of the NLP and FTD factors across groups are provided in the Supplement (Supplementary Table 4).

Associations between NLP speech factors and FTD dimensions were strongest in the psychotic disorder group, particularly for the negative correlations between *Syntactic Complexity* and the FTD dimensions Disorganization, Emptiness, and Incoherence, as well as between *Lexical Diversity and Fluency* and the FTD dimension Emptiness (Supplementary Fig. 4). As a sensitivity analysis, we repeated the correlation analyses excluding participants with psychotic disorders (n = 48) to evaluate whether associations between NLP speech factors and FTD dimensions were driven by this subgroup. The pattern of associations remained largely unchanged, with *Syntactic Complexity* continuing to show significant negative correlations with FTD Disorganization, Emptiness, and Incoherence (Supplementary Analysis S1).

### Associations between NLP speech factors and gray matter volume

**Fig. 1:**
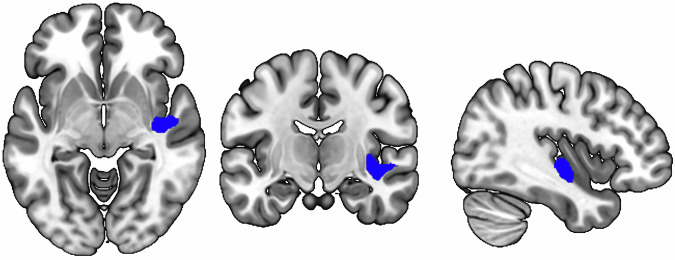
Negative Correlation between NLP Speech Factor *Narrow Thematic Focus* and Gray Matter Volume (*N* = 303). Significant cluster is displayed at a family-wise error-corrected *p*-value < 0.05 at cluster level (initial cluster-defining threshold of *p* < 0.001).

No significant correlations were observed between Factor 1 (Syntactic Complexity) or Factor 2 (Lexical Diversity and Fluency) and GMV after FWE correction. Factor 3 (Narrow Thematic Focus) was negatively associated with the GMV predominantly in the right posterior insula, with additional involvement of the right planum polare and right putamen (see Fig. 1; *k* = 705, x/y/z = 36.0/–15.0/−1.5, *p* = 0.046 FWE; ß = −0.16 with 95% CI [−0.24,−0.09]), after controlling for diagnostic group. This association was not significantly moderated by verbal IQ or medication (Supplementary Table 5).

### Associations between NLP speech factors and diffusion-tensor-imaging

**Fig. 2:**
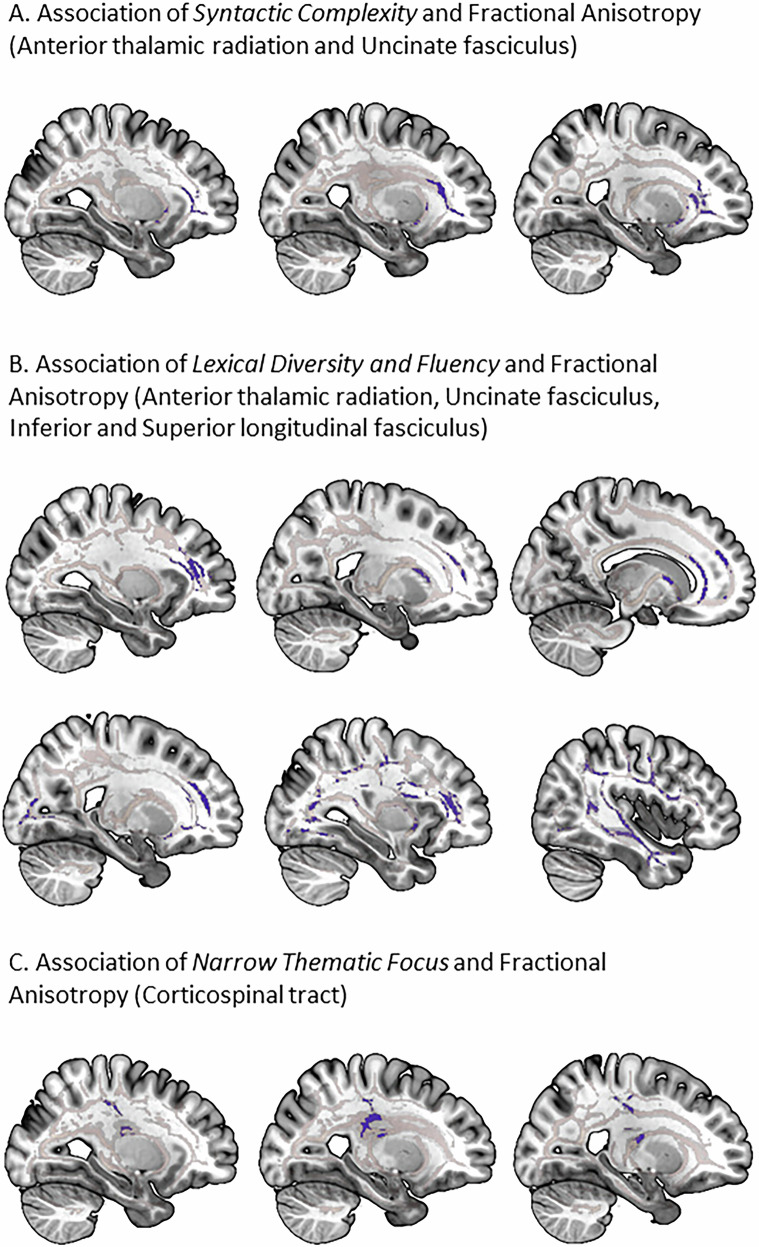
Negative Association between NLP-Derived Speech Factors and White Matter Structure. **A**, **B**, and **C**: Negative Associations between NLP Speech Factors and Fractional Anisotropy in the DTI sample (*N* = 247). Significant clusters are presented at a family-wise error-corrected *p*-value < 0.05.

Significant associations between the NLP-derived speech factors and FA were identified in key white matter tracts, highlighting links between linguistic features and white matter integrity (see Table 4 and Fig. 2). Shared white matter tracts showing significant associations included the left ATR and the left UF, where FA was negatively associated with both *Syntactic Complexity* and *Lexical Diversity and Fluency* (Fig. 2A and B). Additional negative associations for *Lexical Diversity and Fluency* were identified in the right ILF and SLF (Fig. 2B). In the left corticospinal tract (CST), FA exhibited a significant negative association with *Narrow Thematic Focus* (Fig. 2C). Additional moderation analyses revealed that neither verbal IQ nor medication indices significantly moderated these associations (see Supplementary Table 6). Further associations involving AD, RD, and MD revealed similar patterns in frontotemporal tracts, as detailed in Supplementary Table 7.

**Table 4:** Negative Association Between NLP-Derived Speech Factors and Fractional Anisotropy.

Factor	Coordinates of the maximum intensity voxel (x/y/z) MNI	White Matter Tract	Hemisphere	*k*	*P*_FWE_	β
*Syntactic Complexity*	−28/31/12	Anterior thalamic radiation	L	47	0.045	−0.18
	−28/42/−3	Uncinate fasciculus	L	45	0.047	−0.23
*Lexical Diversity and Fluency*	−30/36/11	Anterior thalamic radiation	L	280	0.019	−0.35
	−15/9/4			36	0.028	
	30/38/11	Anterior thalamic radiation	R	149	0.017	−0.33
	21/47/3			43	0.031	
	16/42/−12	Forceps minor	R	93	0.048	−0.23
	22/48/3			45	0.042	
	42/−6/−29	Inferior longitudinal fasciculus	R	1322	**0.016**	−0.27
	34/−60/0			572	0.021	
	34/−69/−9			22	0.045	
	54/−10/22	Superior longitudinal fasciculus	R	632	0.020	−0.30
	37/−24/42			87	0.040	
	−30/36/11	Uncinate fasciculus	L	91	0.040	−0.30
	41/0/−27	Uncinate fasciculus	R	26	0.045	−0.28
	35/0/−18			12	0.049	
*Narrow Thematic Focus*	−28/−23/39	Corticospinal tract	L	85	0.034	−0.21

*N* = 247.

*R* right, *L* left, family-wise-error-corrected *p*-values.

Covariate of no interest included in the regression analyses were age, sex, TIV, and psychiatric diagnoses. Significant results after Bonferroni-correction for the three NLP Speech Factors are in bold.

To contextualize the observed NLP-DTI associations, we conducted a comparative analysis with effect sizes from Stein et al. [2], who reported FA correlates of SAPS/SANS-rated FTD dimension in a large transdiagnostic sample (*N* = 830). Stein et al. found modest negative associations between incoherence and FA in the bilateral ATR (β = −0.15 for each) and a marginal positive association between disorganization and FA in the right ILF (β = 0.03). In contrast, our NLP-derived factor *Diversity and Fluency* showed stronger negative associations with FA in the same tracts (left ATR: β = −0.35; right ATR: β = −0.33, right ILF: ß = −0.27).

To further evaluate the relative sensitivity of NLP-derived versus clinically rated features, we conducted a parallel TBSS analyses using the three FTD factors derived from SAPS and SANS in our current sample (*N* = 242). Results revealed significant negative associations between both Disorganization and Incoherence and FA in the right cingulum bundle of the hippocampal part (see Supplementary Table 8).

## Discussion

This study explored the associations between NLP derived linguistic features of spontaneous speech, FTD, and neuroanatomical structures in a large sample of patients with affective or psychotic disorders, as well as healthy controls. By integrating automated speech analysis with structural and diffusion-weighted neuroimaging, this study offers novel insights into the neural correlates of language disturbance and the methodological advantages of computational linguistic measures. Key findings include the identification of the linguistic dimensions: *Syntactic Complexity*, *Lexical Diversity and Fluency*, and *Narrow Thematic Focus* using exploratory factor analysis of a broad set of NLP-derived syntactic and semantic features. Identified NLP dimensions were correlated with both FTD and brain structure. Hereof, *Syntactic Complexity* was significantly negatively associated with the FTD dimensions Incoherence and Emptiness, while *Lexical Diversity and Fluency* showed a specific negative association with Emptiness, indicating that computational linguistic features correspond to clinically relevant subdomains of thought disorder. Neuroimaging analyses revealed that the *Narrow Thematic Focus* factor was uniquely associated with reduced GMV mainly in the right posterior insular cortex, highlighting a structural correlate of constrained and self-referential speech content. Furthermore, the NLP-derived factors *Syntactic Complexity* and *Lexical Diversity and Fluency* were linked to white matter microstructure within key language-relevant tracts. These associations suggest that language disturbances are supported by disruptions in frontotemporal connectivity. Findings remained stable when correcting for diagnosis, medication, and verbal IQ, supporting a dimensional and transdiagnostic model of language dysfunction in severe mental illness.

### Linguistic factors and formal thought disorder

The NLP-derived factor *Syntactic Complexity*, encompassing grammatical structure and cohesive discourse, correlated negatively with all three FTD dimensions (Disorganization, Emptiness, and Incoherence), consistent with prior research linking syntactic deficits to disordered thinking in psychosis [63, 64] and high-risk individuals [24, 26] as well as in MDD [34, 49]. These results indicate that reduced syntactic complexity reflects a breakdown in the hierarchical and integrative aspects of language production that underpin coherent thought, aligning closely with the clinical constructs of disorganization and incoherence. Our results extend previous work, supporting the notion that decreased *Syntactic Complexity* may serve as a transdiagnostic indicator of disorganization in thought processes, broadening our understanding of FTD as a pervasive feature influencing linguistic expression across diverse mental health conditions.

The NLP factor *Lexical Diversity and Fluency* captured a rich vocabulary, morphological variation, and fluent speech with fewer disfluencies and errors. Its negative correlation with the FTD Emptiness factor suggests that individuals with restricted vocabulary and hesitant speech experience greater poverty of thought content. In this sense, lower scores on *Lexical Diversity and Fluency* capture a quantifiable manifestation of the verbal impoverishment observed clinically as Emptiness, characterized by sparse, repetitive, or vague ideational output. This relationship aligns with prior findings connecting lexical deficits to alogia and impoverished thought [22, 65] and further supports its utility as an early indicator of psychosis [66, 67] and cognitive narrowing in the prodromal phases [68, 69]. Moreover, recent studies have linked alterations in lexical content with depressive states [70–72]. The present study reinforces the importance of lexical variability as a transdiagnostic indicator of cognitive and linguistic processes in psychiatric conditions.

The NLP factor *Narrow Thematic Focus*, characterized by frequent pronoun use and high semantic density, was not significantly correlated with any of the clinician-rated FTD dimensions. This lack of association may reflect that Narrow Thematic Focus captures a form of speech restriction centered on repetitive, self-referential, or topically constrained narratives, which can maintain local coherence and logical form despite reduced narrative variability. As such, it may represent a distinct linguistic style not directly indexed by conventional FTD ratings of the SANS and SAPS scales, which primarily assess disruptions in logic and global coherence rather than thematic restriction. More detailed FTD scales like the TALD might have shown different results.

Our findings contribute to a growing body of evidence supporting the dimensional approach to studying FTD across diagnoses, emphasizing the shared nature of language impairments in psychiatric conditions. For instance, Stein et al. [2] identified three core dimensions of FTD—disorganization, emptiness, and incoherence—validated across MDD, BD, and SSD. Tang et al. [15] similarly confirmed a comparable structure, reinforcing the transdiagnostic nature of FTD. In the present study, this factor model was once again confirmed. Moreover, our NLP-derived speech factors also demonstrated transdiagnostic patterns. Thus, our findings underscore the value of automated speech analysis as a tool to objectively assess language disturbances across diagnoses. Notably, language disturbances, including FTD, are prevalent not only in SSD but also in affective disorders, further supporting their role as transdiagnostic features of psychopathology [1, 2, 10, 13, 16, 73].

### Neural correlates of language disturbances

The present study underscores a transdiagnostic framework, demonstrating that linguistic dimensions are linked to shared brain structure independent of diagnosis, current medication, and verbal IQ. Importantly, the observed associations between white matter tracts and NLP-derived speech features were not only anatomically widespread but also of greater magnitude compared to both (a) FTD ratings from Stein et al.’s [2] sample and (b) FTD factor scores derived from the current sample. This suggests that computational measures may be more sensitive to fine-grained disruptions in language–brain coupling than human ratings, possibly reflecting the limits of existing clinical scales and/or human raters in capturing the full spectrum of language abnormalities.

Specifically, FA in the ATR was strongly negatively correlated with the NLP speech factors *Syntactic Complexity* as well as *Lexical Diversity and Fluency*. The ATR, which connects the thalamus with the prefrontal cortex, plays a critical role in memory and language processing [74, 75], and ATR abnormalities have been linked to deficits in cognitive abilities and psychopathology [76, 77].

In addition, significant correlations were observed between NLP speech factors and FA in additional key white matter tracts, such as the UF and the right SLF. The UF connects the temporal pole with the frontal lobe and is involved in semantic processing and the integration of emotional and cognitive information [78, 79], while the SLF facilitates interhemispheric communication and is implicated in language processing, attention, and working memory [79, 80]. These findings align with previous reports of altered FA in these regions in both psychosis [40, 81–83] and affective disorders [83–86]. *Narrow Thematic Focus* and FA were negatively associated in the left CST. Although the CST is primarily a motor pathway, its recognized involvement in prosody and articulatory control [87, 88], suggesting motor pathway integrity may also influence language production.

In addition to associations with FA, significant correlations were also observed between NLP-derived speech factors and AD, RD, and MD in frontotemporal white matter tracts. This represents a methodological extension of prior work such as that by de Boer et al. [47], who focused exclusively on FA and MD in SZ. By applying a transdiagnostic framework and integrating multiple DTI parameters, the current study offers a more comprehensive characterization of white matter correlates of spontaneous speech across psychiatric populations.

Beyond white matter findings, neuroimaging analyses identified an association between *Narrow Thematic Focus* and reduced GMV in a cluster centered in the right posterior insula, with additional contributions from the right putamen and the right planum polare. The insula plays a critical role in language processing [89, 90]. The planum polare, located in the superior temporal gyrus, is often implicated in auditory processing and may also be involved in aspects of language comprehension [91]. The putamen, a subcortical component of the dorsal striatum within the basal ganglia, is involved in motor control and has further been associated with aspects of speech production and articulatory control [92, 93]. Moreover, our findings of altered GMV in the insula and putamen corroborates previous reports in psychotic disorders [94–96], as well as in affective disorders [97–100].

Methodologically, this study advanced the field by integrating state-of-the-art NLP techniques with neuroimaging in a transdiagnostic sample. Whereas prior work [49] relied on manually coded syntax, we applied exploratory factor analysis to a comprehensive set of computationally derived linguistic features to identify latent dimensions of spontaneous speech. As the first study of its kind, this approach offers a more holistic view of linguistic features, capturing, for example, both aspects of Schneider’s [34] syntactic complexity and diversity and provide a more sensitive assessment of differences in language performance across diagnostic groups. Prior NLP studies in SZ have linked coherence and semantic similarity to structural or functional abnormalities in frontotemporal circuits [45–48]. However, those studies were restricted to SZ and typically focused on isolated linguistic markers. In contrast, our study demonstrates that (1) similar language–brain associations extend across diagnostic categories and (2) these associations are more comprehensively captured using multivariate, latent speech constructs derived from NLP.

Together, our findings support a dimensional, transdiagnostic framework of language disturbance, challenging traditional categorical approaches to psychiatric diagnosis. In line with the Research Domain Criteria (RDoC) framework [101], our results demonstrate that language impairments and their neural correlates are not confined to specific diagnoses but reflect shared disruptions across psychotic and affective disorders. Critically, our findings highlight the added value of automated speech analysis over traditional psychopathological assessments. These findings are consistent with prior evidence showing that automated speech analysis offers significant advantages by objectively quantifying subtle language disruptions that may otherwise go undetected in traditional clinical assessments [24, 30, 66, 102].

Nevertheless, some limitations should be acknowledged. First, our analyses were conducted in German, which raises questions of cross-linguistic generalizability. While some speech markers, particularly those related to semantic coherence are likely to generalize across languages, others, such as pronoun use or syntactic complexity, may vary depending on grammatical structure. Future cross-linguistic validation in languages with differing morphological and syntactic properties will be essential for establishing their universality. Second, the cross-sectional design precludes conclusion about causality. Longitudinal studies are necessary to determine whether language abnormalities precede, follow, or co-develop with neuroanatomical alterations. Third, although our sample size was large, imbalances across diagnostic groups may affect the generalizability of the findings, a limitation that was partially mitigated by statistically controlling for diagnostic category in all analyses. Finally, the use of TAT-based speech elicitation, while standardized, may not fully capture the complexity of real-world language use. Incorporating more naturalistic speech paradigms, such as free conversation or dialogic tasks may enhance the ecological validity of linguistic assessments in future studies.

## Conclusion

This study demonstrates that linguistic features extracted from spontaneous speech using NLP techniques are significantly associated with FTD symptoms and structural brain abnormalities in a large transdiagnostic sample. The identified NLP speech factors—Syntactic Complexity, Lexical Diversity and Fluency, and Narrow Thematic Focus — provide valuable insights into the linguistic impairments in individuals with psychiatric disorders and healthy controls. The findings specifically highlight the associations between linguistic factors and structural brain abnormalities, emphasizing the intricate relationship between language processing capabilities and neural infrastructure. By capturing subtle, clinically relevant language disruptions, automated methods provide a powerful tool for examining language disturbances in greater detail. This approach advances our understanding of the complex interplay between language and spontaneous speech pathology.

## Supplementary information

Supplementary Information for Linking Speech Patterns and Brain Structure

## Data Availability

The data supporting the findings of this study can be accessed by contacting the corresponding author. All code used for NLP-based speech feature extraction and exploratory factor analysis is available at https://github.com/NeuroSTA/NeuroSTA.
