# Supplementary material for: Linking speech patterns to brain structure in affective and psychotic disorders: an integrative natural language processing approach
Source: Mol Psychiatry. 2025 Nov 20;31(4):2057–68. doi: 10.1038/s41380-025-03347-9 (PMC12999474; doi:10.1038/s41380-025-03347-9)
Supplement: Supplementary file 1 — Supplementary Information for Linking Speech Patterns and Brain Structure [file 41380_2025_3347_MOESM1_ESM.docx]

**Supplement**

## ****eMethod 1. Extraction of NLP-derived speech features****

**Participants were presented with four images from the Thematic Apperception Test (TAT) and asked to narrate a story for three minutes per image, yielding approximately 12 minutes of spontaneous speech per participant. The extended time window was chosen to allow for the emergence of complex speech characteristics, such as FTD-related disruptions, that might not manifest in shorter narratives. Participants were given a one-minute break between stories, during which the instructions were repeated. If participants stopped during the three-minute window, up to two non-directive prompts (e.g., “How do people feel?”; “What could happen next?”) were provided to encourage continued speech production.** **All participants were native German speakers. Speech samples were recorded using an Olympus WS-853 digital recorder and transcribed verbatim by trained linguists using the f4transkript software (https://www.audiotranskription.de/f4transkript). Transcribers were blinded to participants' formal diagnoses to avoid bias. Transcriptions included all verbal utterances, including disfluencies such as pauses, fillers, and repetitions, to ensure a comprehensive capture of speech characteristics. All speech transcripts were preprocessed before feature extraction. The preprocessing steps included the removal of non-linguistic content, such as speaker identifiers and annotation comments, followed by tokenization and segmentation into sentences using spaCy’s German library model "de_dep_news_trf" [1].** Sentence boundaries were determined via spaCy’s dependency parsing–based segmentation (doc.sents). **The spacy library (version 3.5.2) [2] was used for NLP.**

**Because some linguistic measures can be affected by transcript length, we applied strategies to ensure comparability across participants. Lexical diversity was assessed using the Type–Token Ratio (TTR) and the Measure of Textual Lexical Diversity (MTLD), with the latter providing a length-independent and more stable estimate across transcripts of varying sizes. Count-based measures—such as pronoun usage, connective frequency, grammatical error rate, and filled pauses—were normalized by expressing them as ratios relative to total words, sentences, or clauses. Semantic features derived from FastText, BERT, and spaCy embeddings, as well as semantic density measures, were calculated using cosine similarity, which is inherently independent of overall text length.**

Lexical features included the Type-Token Ratio (TTR), assessing lexical diversity by dividing the number of unique words by the total number of words in the transcript. The Measure of Textual Lexical Diversity (MTLD) was calculated to provide a stable assessment of lexical diversity across texts of varying lengths [3]. The MTLD accounts for the rate at which new vocabulary is introduced in the text, offering a more reliable measure of lexical diversity for longer transcripts. Pronoun usage was assessed by calculating the overall pronoun ratio, which is the proportion of pronouns to total tokens, reflecting reference to entities within the speech. Additionally, the personal pronoun ratio was determined by calculating the proportion of personal pronouns to total pronouns, indicating self-references. For the these metrics and the Part-of-speech (POS) ratios, the spaCy library [2] and the German spacy model “de_dep_news_trf” [1] were used.

Morphological features involved analyzing morphological complexity, measuring the use of inflections and derivations in speech. Morphological features were extracted using spaCy's morphological analysis capabilities, providing insights into cognitive or linguistic impairments [4].

Syntactic features encompassed measures such as syntactic complexity, a combined measure including average sentence length and the depth of syntactic dependency trees generated using spaCy's dependency parser. The subordination ratio, reflecting the proportion of subordinate clauses to the total number of clauses, was calculated to assess sentence complexity. The use of connectives was extracted via the connective ratio per sentence to assess discourse cohesion. The readability score was computed using the Hohenheim Index, a readability metric specific to the German language, which combines factors like sentence length and syllables per word to indicate text difficulty and cognitive load [5, 6]. Higher values on this index correspond to easier readability, suggesting simpler sentence constructions.

Several semantic features were employed to capture different aspects of semantic processing in speech. By employing these diverse semantic measures, we aimed to capture both the local coherence between sentences and the global semantic properties of the speech, providing a comprehensive analysis of semantic processing in participants' narratives. Semantic coherence was assessed by computing the cosine similarity between consecutive sentences using word embeddings from the German fastText model “cc.de.300.bin” [7]. FastText captures word-level semantics, including subword information, allowing for an assessment of how semantically related adjacent sentences are, reflecting the speaker's ability to maintain thematic continuity. Sentence-level coherence was assessed using Bidirectional Encoder Representations from Transformers (BERT) embeddings from a pre-trained German BERT model “bert-base-german-cased” [8], capturing deeper contextual relationships between sentences. BERT embeddings consider the entire context of a sentence, enabling the assessment of more nuanced coherence, such as subtle shifts in topic or the introduction of new concepts. Word-level coherence was assessed using spaCy's word vectors to compute semantic coherence across all words. It provides a granular view of how individual words semantically relate to each other throughout the speech, capturing the consistency of semantic themes and lexical choices. Semantic density was measured by computing the average pairwise semantic similarity between content words within the entire transcript. It reflects the overall semantic richness and specificity of the speech by assessing how semantically related the content words are throughout the narrative.

Graph-based cohesion was evaluated by computing the average shortest path length in word graphs, reflecting connectivity and cohesion in speech [9]. In these graphs, words were represented as nodes, and edges were established based on word co-occurrence. Disfluency measures included the filled pauses ratio, reflecting the frequency of filler words such as "um" and "ah" relative to total words, indicating hesitancy or interruptions in speech. The word repetition frequency was computed as the ratio of repeated words to total words. The grammatical error ratio was assessed by calculating the proportion of grammatical errors to total words, evaluating deviations from standard language use. Sentiment Analysis was conducted using a model trained on German text (“german-sentiment-bert”) [10] to estimate the probability of negative sentiment in the speech, reflecting the emotional tone of the speech.

Control analyses for syntactic complexity and verbosity-related measures

Because syntactic complexity is mathematically related to utterance length, we evaluated whether this feature was confounded by verbosity-related aspects of speech. Specifically, we tested the raw syntactic complexity variable (average subordination depth per sentence) against measures of verbosity, including mean length of utterance (MLU), total word count, and number of sentences, as well as against the FTD Emptiness factor and the SANS item “poverty of speech.”

As expected, syntactic complexity showed a strong correlation with MLU (r = .77, p < .001) and a moderate correlation with total word count (r = .48, p < .001), as well as a negative correlation with the number of sentences (r = −.37, p < .001). This pattern reflects the inherent mathematical dependency between clausal subordination and utterance length. Importantly, however, syntactic complexity did not correlate with clinician-rated poverty of speech (r = −.09, p = .088) and showed only a modest association with the FTD Emptiness factor (r = −.17, p = .001), suggesting that it does not simply index speech quantity or clinical ratings of reduced output.

## ****eMethod 2. Neuroimaging Data Acquisition and Preprocessing****

A 12-channel head matrix receive coil was used for all scans. High-resolution T1-weighted images were obtained using a fast gradient-echo magnetization-prepared rapid acquisition gradient echo (MP-RAGE) sequence. The imaging parameters were: repetition time (TR) = 1,900 ms; echo time (TE) = 2.26 ms; inversion time (TI) = 900 ms; flip angle = 9°; field of view (FOV) = 256 mm; slice thickness = 1.0 mm; 176 sagittal slices; and a voxel size of 1 × 1 × 1 mm³.

DTI data was acquired using an echo-planar imaging (EPI) sequence. The imaging parameters were: TR = 7,300 ms; TE = 90 ms; FOV = 320 mm; phase encoding direction = anterior-posterior; 56 slices with a slice thickness of 3 mm, resulting in a voxel resolution of 2.5 × 2.5 × 2.5 mm³. Two sets of diffusion-weighted images were collected, each with 30 diffusion-weighted directions at a b-value of 1,000 s/mm², along with four non-diffusion-weighted images (b = 0 s/mm²).

Structural MRI data were processed using the default parameters of the Computational Anatomy Toolbox (CAT12, v1720) [11](CAT12; Gaser et al., 2024) implemented within the Statistical Parametric Mapping software SPM12 (v7771; Structural Brain Mapping Group at Jena University Hospital, Germany, available at <http://dbm.neuro.uni-jena.de/cat/>). Images were spatially registered, segmented into gray matter, white matter, and cerebrospinal fluid compartments, and normalized to the Montreal Neurological Institute (MNI) space using DARTEL normalization. The normalized gray matter images were smoothed with an 8 mm full-width at half-maximum (FWHM) Gaussian kernel to accommodate inter-individual anatomical variability and enhance the signal-to-noise ratio. Total intracranial volume (TIV) was calculated during preprocessing.

For the DTI analyses, a tract-based spatial statistics (TBSS) approach was applied using the FMRIB Software Library (FSL) [12]. Preprocessing was conducted using default parameters, including correction for motion and Eddy-Current artifacts. The data were first aligned to the standard MNI space through non-linear registration, using an FSL template. FA maps were generated and subsequently projected onto a mean FA skeleton representing the central pathways of the tracts common to all participants. A threshold of 0.2 was applied to the FA skeleton to minimize misalignment errors and ensure precise voxel-wise statistical comparisons.

**Supplementary Table 1 and Supplementary Table 2 provide the descriptive statistics for the final GMV (N = 303) and TBSS/DTI (N = 247) analytic samples, respectively.**

### ****Supplementary Table 1.** Descriptive Statistics of the GMV Cluster Analysis Sample (*N* = 303)**

| Variable | Healthy controls  (*n* = 150) | Affective disorders  (*n* = 110) | Psychotic disorders  (*n* = 43) | *p* | Effect size |
| --- | --- | --- | --- | --- | --- |
| **Age** | 43.87 (13.53) | 43.35 (12.45) | 40.72 (12.52) | .378 |  |
| Sex, *n* (f/m) | 95/55.00 | 66/44.00 | 16/27.00 | **.008** | **V = .18** |
| **Years of education** | 14.63 (3.23) | 13.41 (2.89) | 12.00 (2.25) | **< .001 ^a,b,c^** | η² = .09 |
| IQ | 116.21 (12.90) | 115.12 (14.38) | 111.37 (14.90) | .140 |  |
| **TIV** | 1530.03 (143.51) | 1551.19 (153.63) | 1565.62 (155.08) | .294 |  |
| **SANS nFTD subscale** | 0.26 (0.78) | 1.22 (2.06) | 2.51 (2.89) | **< .001^d,e,f^** | η² = .17 |
| **SAPS pFTD subscale** | 0.47 (1.21) | 1.8 (3.26) | 5.67 (7.63) | **< .001^d,e,f^** | η² = .19 |
| **SANS sum** | 1.41 (2.87) | 8.18 (8.70) | 17.3 (12.17) | **< .001^d,e,f^** | η² = .37 |
| **SAPS sum** | 0.52 (1.31) | 2.19 (3.75) | 12.6 (13.22) | **< .001^d,e,f^** | η² = .35 |
| **YMRS sum** | 0.43 (1.27) | 1.22 (3.26) | 5.83 (6.95) | **< .001^d,e,f^** | η² = .22 |
| **HAM-D sum** | 1.39 (2.23) | 5.57 (6.02) | 8.53 (7.48) | **< .001^d,e,f^** | η² = .23 |
| **HAMA-A sum** | 1.84 (2.64) | 7.41 (7.13) | 10.03 (8.34) | **< .001^d,e,f^** | η² = .25 |
| **GAF score** | 85.98 (8.77) | 65.71 (13.39) | 52.92 (15.35) | **< .001^a,b,c^** | η² = .54 |

***Note.*  Mean (standard deviation); TIV: total intracranial volume; SANS: scale for the assessment of negative symptoms for the assessment of positive symptoms; SAPS: scale for the assessment of positive symptoms; nFTD = negative formal thought disorder; pFTD = positive formal thought disorder; YMRS: Young mania rating scale; HAM-D: Hamilton rating scale for depression; HAM-A: Hamilton rating scale for anxiety; GAF: global assessment of functioning; η²** represents **Eta-Squared**, a measure of effect size in ANOVA that quantifies the proportion of variance in the dependent variable attributable to group differences; **V represents Cramér's V, a measure of effect size for Chi-square tests of independence that indicates the strength of association between categorical variables; significant results after Bonferroni correction for multiple testing are in bold**

^a^ =  Affective disorders < Healthy controls

^b^ =  Psychotic disorders < Healthy controls

^c^ =  Psychotic disorders < Affective disorders

^d^ =  Affective disorders > Healthy controls

^e^=  Psychotic disorders > Healthy controls

^f^ =  Psychotic disorders > Affective disorders

### ****Supplementary Table 2.** Descriptive Statistics of the White Matter Tract Analysis Sample (*N* = 247)**

| Variable | Healthy controls  (*n* = 121) | Affective disorders  (*n* = 92) | Psychotic disorders  (*n* = 34) | *p* | Effect size |
| --- | --- | --- | --- | --- | --- |
| **Age** | 43.83 (13.34) | 42.48 (12.17) | 40.5 (12.17) | .378 |  |
| Sex, *n* (f/m) | 80/41 | 55/37 | 13/21 | **.014** | **V = .19** |
| **Years of education** | 14.6 (3.07) | 13.12 (2.68) | 11.74 (2.11) | **< .001 ^a,b,c^** | η² = .12 |
| IQ | 116.15 (13.08) | 115.13 (14.49) | 109.59 (14.85 | .061 |  |
| **TIV** | 1522.5 (145.76) | 1562.54 (157.55) | 1548.92 (138.15) | .147 |  |
| **SANS nFTD subscale** | 0.31 (0.86) | 0.93 (1.59) | 1.97 (2.44) | **< .001^d,e,f^** | η² = .13 |
| **SAPS pFTD subscale** | 0.44 (1.18) | 1.82 (3.39) | 4.59 (6.2) | **< .001^d,e,f^** | η² = .16 |
| **SANS sum** | 1.43 (2.88) | 7.35 (6.9) | 15.84 (12.37) | **< .001^d,e,f^** | η² = .37 |
| **SAPS sum** | 0.49 (1.24) | 2.07 (3.72) | 12.62 (13.32) | **< .001^d,e,f^** | η² = .35 |
| **YMRS sum** | 0.48 (1.37) | 0.99 (2.75) | 5.52 (7.22) | **< .001^d,e,f^** | η² = .21 |
| **HAM-D sum** | 1.44 (2.32) | 5.63 (6.23) | 8.47 (7.64) | **< .001^d,e,f^** | η² = .22 |
| **HAMA-A sum** | 1.98 (2.77) | 7.17 (7.07) | 9.47 (8.18) | **< .001^d,e,f^** | η² = .22 |
| **GAF score** | 85.79 (8.77) | 66.14 (13.43) | 54.34 (15.51) | **< .001^a,b,c^** | η² = .51 |

***Note.*  Mean (standard deviation); TIV: total intracranial volume; SANS: scale for the assessment of negative symptoms for the assessment of positive symptoms; SAPS: scale for the assessment of positive symptoms; nFTD = negative formal thought disorder; pFTD = positive formal thought disorder; YMRS: Young mania rating scale; HAM-D: Hamilton rating scale for depression; HAM-A: Hamilton rating scale for anxiety; GAF: global assessment of functioning; η²** represents **Eta-Squared**, a measure of effect size in ANOVA that quantifies the proportion of variance in the dependent variable attributable to group differences; **V represents Cramér's V, a measure of effect size for Chi-square tests of independence that indicates the strength of association between categorical variables; significant results after Bonferroni correction for multiple testing are in bold**

^a^ =  Affective disorders < Healthy controls

^b^ =  Psychotic disorders < Healthy controls

^c^ =  Psychotic disorders < Affective disorders

^d^ =  Affective disorders > Healthy controls

^e^=  Psychotic disorders > Healthy controls

^f^ =  Psychotic disorders > Affective disorders

## ****eMethod 3. Statistical Analysis****

**Statistical analyses were conducted using R (version 4.4.1) [13].**

**Exploratory Factor Analysis (EFA)**

To reduce dimensionality and identify latent linguistic constructs, an EFA was performed of the NLP-derived speech features. The adequacy of the feature correlation matrix was confirmed using the Kaiser-Meyer-Olkin (KMO) measure of sampling adequacy and Bartlett’s test of sphericity. EFA was conducted using the Unweighted Least Squares (ULS) estimation method, which is robust to deviations from multivariate normality. Promax rotation was employed to allow for correlated factors. This method facilitates a more interpretable solution by reflecting real-world relationships among variables more accurately [14]. Research indicates that promax rotation can yield better-fitting models in situations where factors are not independent, enhancing the clarity of factor interpretation [15]. The number of factors to retain was determined using multiple complementary criteria, including the empirical Kaiser criterion [16], the Hull method [17], and the scree plot inspection [18], to robustly support the factor solution.

An average factor solution was derived using the “EFA_AVERAGE” function from EFAtools [19], which aggregates solutions across multiple extraction methods (Principal Axis Factoring, Maximum Likelihood, and Unweighted Least Squares). This procedure enhances generalizability by reducing dependence on a single estimation technique.

EFA analyses were conducted using the EFAtools [19] and the psych packages [20].

**Confirmatory Factor Analysis (CFA)**

To evaluate the validity of the FTD structure reported in previous work [21], we conducted a CFA on SAPS and SANS items. Model estimation used robust maximum likelihood (MLR) to account for deviations from normality. Model specification and estimation were conducted using the lavaan package [20]. Model fit was evaluated using the comparative fit index (CFI), the Tucker-Lewis index (TLI), and the root mean square error of approximation (RMSEA).

## eResults 1. Exploratory Factor Analysis of NLP Speech Features

An exploratory factor analysis was conducted on the NLP-derived speech features to reduce dimensionality and identify latent linguistic constructs. Data adequacy was confirmed by a high Kaiser–Meyer–Olkin value and a significant Bartlett’s test of sphericity, indicating that the correlation matrix was well suited for factor analysis. In addition to examining the intercorrelation structure, variance inflation factors (VIFs) were calculated for all features to assess multicollinearity, and all values were below 5, indicating an absence of problematic redundancy among predictors. The heatmap in Supplementary Figure 1 visualizes the full intercorrelation structure among the extracted features, revealing distinct clusters of strongly associated variables that align with the final factor solution. Bootstrapped loadings demonstrated high stability of the factor structure. Supplementary Figure 2 depicts the average factor-loading patterns across the three extraction methods (Principal Axis Factoring, Maximum Likelihood, and Unweighted Least Squares, each with oblique rotation), with factor identities remaining stable across approaches, providing additional support for the reliability and interpretability of the latent structure.

The distribution of the three NLP-derived speech factors across healthy controls, affective disorder patients, and psychotic disorder patients is presented in Supplementary Figure 3. Violin plots show the full score distribution, with overlaid box plots indicating median values and interquartile ranges. Significant group differences emerged for Syntactic Complexity and Lexical Diversity and Fluency, with both healthy controls and affective disorder patients scoring significantly higher than psychotic disorder patient. No significant group differences were found for Narrow Thematic Focus.

### Supplementary Figure 1. Correlation Matrix of the Extracted NLP-Derived Speech Features

**
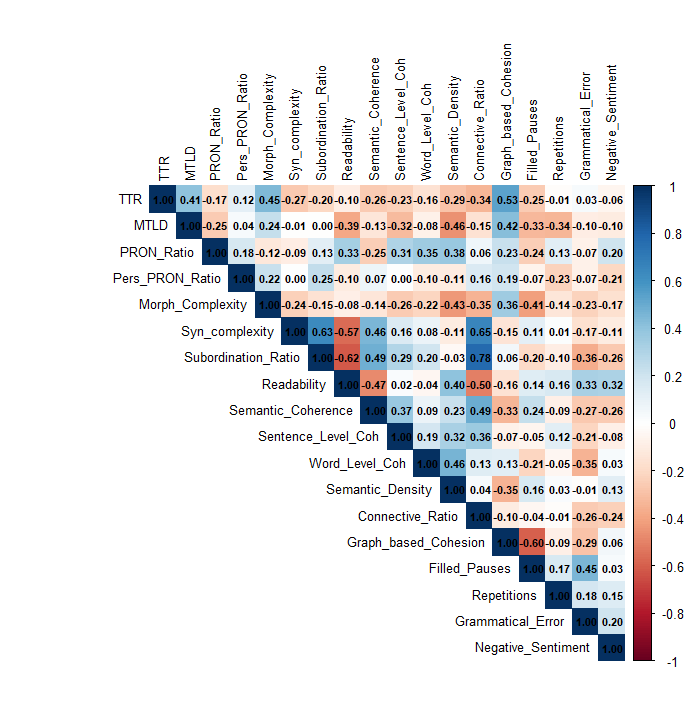
**

### **Supplementary Figure 2. Average Solutions of NLP-Derived Speech Factor Loadings Across Different EFA Methods (F1 = *Syntactic Complexity*, F2= *Lexical Diversity and Fluency*, F3 = *Narrow Thematic Focus*)**


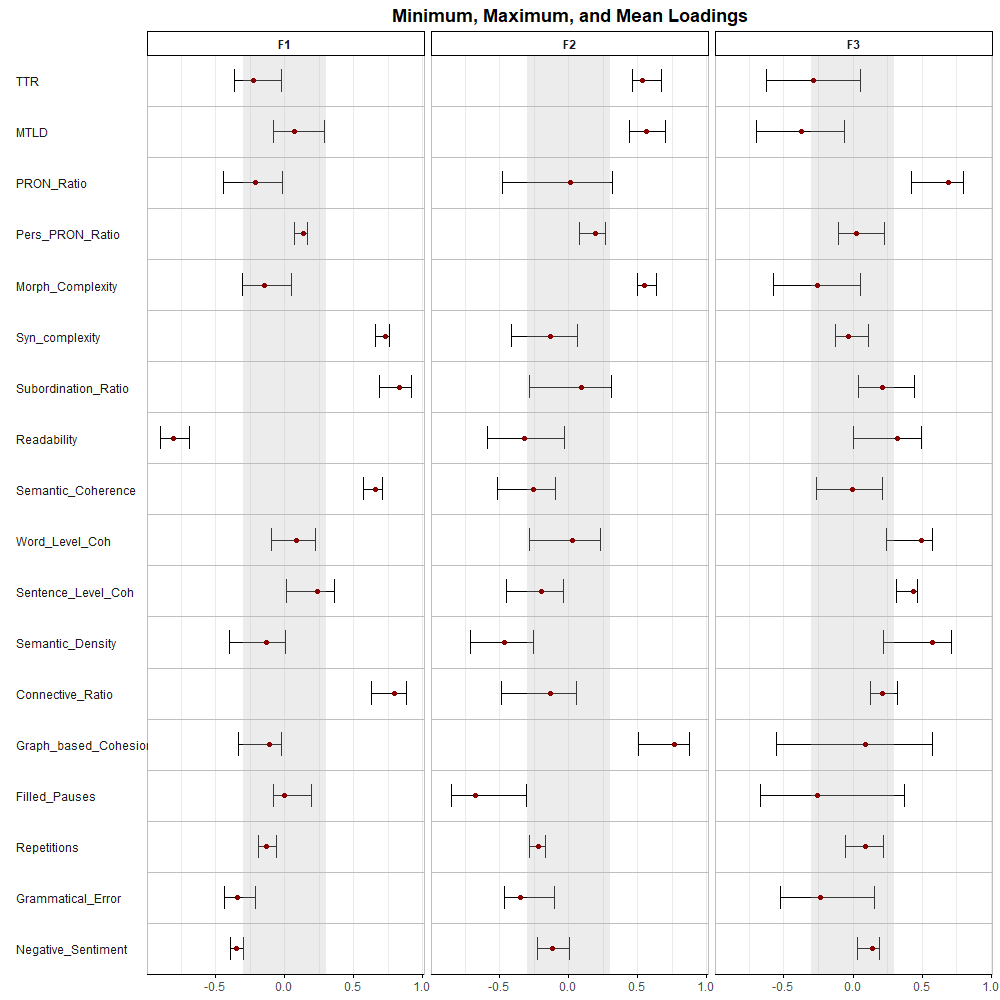


***Notes*. Different extraction methods included** **Principal Axis Factoring (PAF), Maximum Likelihood (ML), and Unweighted Least Squares (ULS), all with oblique rotations.**

### **Supplementary Figure 3. Distribution of NLP Speech Factor Scores Across Groups**


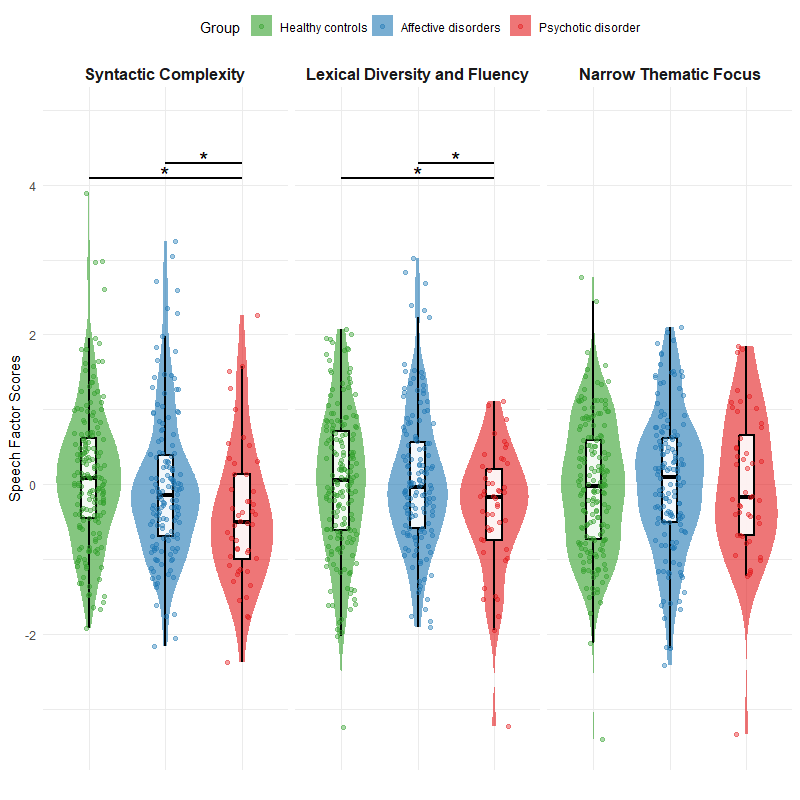


## eResults 2. Confirmatory Factor Analysis of FTD Items

**A confirmatory factor analysis (CFA) was conducted on the SAPS and SANS items assessing formal thought disorder to evaluate the factor structure reported in previous work [21, 22]. The analysis supported a three-factor model comprising Disorganization, Emptiness, and Incoherence, consistent with earlier studies . Model fit indices indicated an acceptable fit to the data, χ²(41) = 95.96, p < .001, CFI = 0.916, TLI = 0.887, RMSEA = 0.061, and SRMR = 0.078. Notably, none of the participants in the present sample were included in the cohort analyzed by Stein et al. [21], ensuring that the current CFA represents an independent validation.**

**The factor composition closely mirrored prior reports, confirming the interpretability and applicability of the three dimensions for subsequent analyses. Standardized factor loadings, along with standard errors and McDonald’s Omega reliability coefficients, are provided in Supplementary Table 3. Reliability indices indicated satisfactory internal consistency for the Disorganization and Incoherence dimensions, with somewhat lower reliability for Emptiness, in line with prior findings.**

### ****Supplementary Table 3.** Results From a Confirmatory Factor Analysis of Format Thought Disorder (FTD) related SAPS and SANS items**

| Factor | Item | Loading | SE | ω |
| --- | --- | --- | --- | --- |
| Disorganization | circumstantiality | **.783** | .083 | .890 |
|  | derailment | **.697** | .090 |  |
|  | tangentiality | **.660** | .075 |  |
|  | pressure of speech | **.631** | .078 |  |
| Emptiness | increased latency of response | **.481** | .131 | .606 |
|  | poverty of content | **.480** | .103 |  |
|  | poverty of speech | .242 | .096 |  |
|  | blocking | .183 | .069 |  |
| Incoherence | illogicality | **.535** | .089 | .770 |
|  | incoherence | .339 | .097 |  |
|  | distractibility | .331 | .078 |  |

***Note.*  *N* = 364. The extraction method was unweighted least squares with an oblique (Promax) rotation. Factor loadings above .40 are in bold. ω = Reliability index McDonald's Omega; SANS: Scale for the Assessment of Negative Symptoms; SAPS: Scale for the Assessment of Positive Symptoms**

## **eResults 3.** Association between NLP Speech factors and FTD Factors

To complement the main transdiagnostic analyses, we provide detailed group-level results here. Descriptive statistics for NLP-derived factors and clinician-rated FTD dimensions by diagnostic group are presented in Supplementary Table 4. Effect size estimates (η²) indicated small-to-moderate group differences across both speech-derived and clinical dimensions. Post-hoc comparisons showed that patients with psychotic disorders scored significantly lower than both healthy controls and patients with affective disorders on the NLP factors Syntactic Complexity and Lexical Diversity and Fluency. Conversely, psychotic disorder patients exhibited significantly higher scores on all three FTD dimensions (Disorganization, Emptiness, Incoherence).

The associations between NLP factors and FTD dimensions across groups are visualized in Supplementary Figure 4. Panels A–C display the negative relationships between Syntactic Complexity and the FTD dimensions Disorganization, Emptiness, and Incoherence. Panel D illustrates the association between Lexical Diversity and Fluency and the FTD dimension Emptiness. Across all significant associations, higher symptom severity was linked to reduced syntactic complexity or diminished lexical diversity and fluency. These associations were most pronounced in the psychotic disorder group, while healthy controls generally clustered toward higher NLP factor scores and lower FTD severity. To assess whether our main findings generalize beyond this subgroup, we repeated the correlation analyses in a subsample excluding all participants with schizophrenia spectrum disorders (see Supplementary Analysis 1). The results demonstrate that most of the observed relationships between NLP-derived speech features and FTD are not solely driven by participants with psychotic disorders, but also hold in a sample restricted to affective disorders and healthy controls.

To further improve interpretability, we additionally examined correlations between individual linguistic features and FTD items of the SANS and SAPS scales (see Supplementary Figure 5). After correction for multiple comparisons, significant effects were confined to a limited set of syntactic and coherence-related measures: syntactic complexity, subordination ratio, semantic coherence, and connective ratio were negatively associated with circumstantiality, while only syntactic complexity and subordination ratio showed reliable correlations with derailment. The inclusion of single-feature analyses provides readers with both dimensional and feature-level perspectives, thereby enhancing interpretability while guarding against overinterpretation of potentially spurious findings.

### ****Supplementary Table 4.** Distribution of the NLP-Derived Speech Factors and Formal Thought Disorder (FTD) Factors**

| Variable | Healthy controls  (*n* = 178) | Affective disorders  (*n* = 146) | Psychotic disorders  (*n* = 48) | *p* | η² |
| --- | --- | --- | --- | --- | --- |
| ***Syntactic Complexity*** | 0.12 (0.93) | -0.02 (0.98) | -0.39 (0.94) | **.004 ^b,c^** | .03 |
| ***Lexical Diversity and Fluency*** | 0.04 (0.96) | 0.07 (0.93) | -0.34 (0.83) | .026 ^b,c^ | .02 |
| ***Narrow Thematic Focus*** | -0.04 (0.89) | 0.05 (0.92) | 0.00 (0.97) | .713 |  |
| **FTD Disorganization** | -0.28 (0.37) | 0.00 (0.83) | 1.07 (1.82) | **< .001 ^d,e,f^** | .20 |
| FTD Emptiness | -0.31 (0.32) | 0.07 (0.81) | 0.94 (1.37) | **< .001 ^d,e,f^** | .23 |
| FTD Incoherence | -0.25 (0.21) | -0.03 (0.80) | 1.05 (1.91) | **< .001 ^d,e,f^** | .19 |

***Note.* Mean (standard deviation). η²** represents **Eta-Squared**, a measure of effect size in ANOVA that quantifies the proportion of variance in the dependent variable attributable to group differences; **significant results after Bonferroni correction for multiple testing are in bold**

^a^ =  Affective disorders < Healthy controls

^b^ =  Psychotic disorders < Healthy controls

^c^ =  Psychotic disorders < Affective disorders

^d^ =  Affective disorders > Healthy controls

^e^=  Psychotic disorders > Healthy controls

^f^ =  Psychotic disorders > Affective disorders

### ****Supplementary Figure 4.** Association Between NLP-Derived Speech Factors and FTD Factors Across Groups**

1. **Association of** Syntactic Complexity **and FTD Factor Disorganization**

**
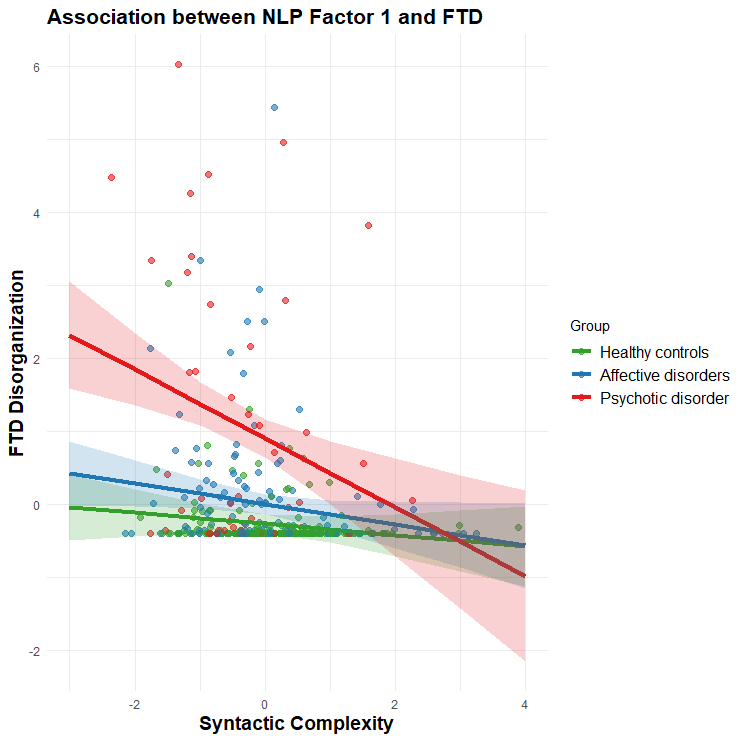
**

1. **Association of** Syntactic Complexity **and FTD Factor Emptiness**

**
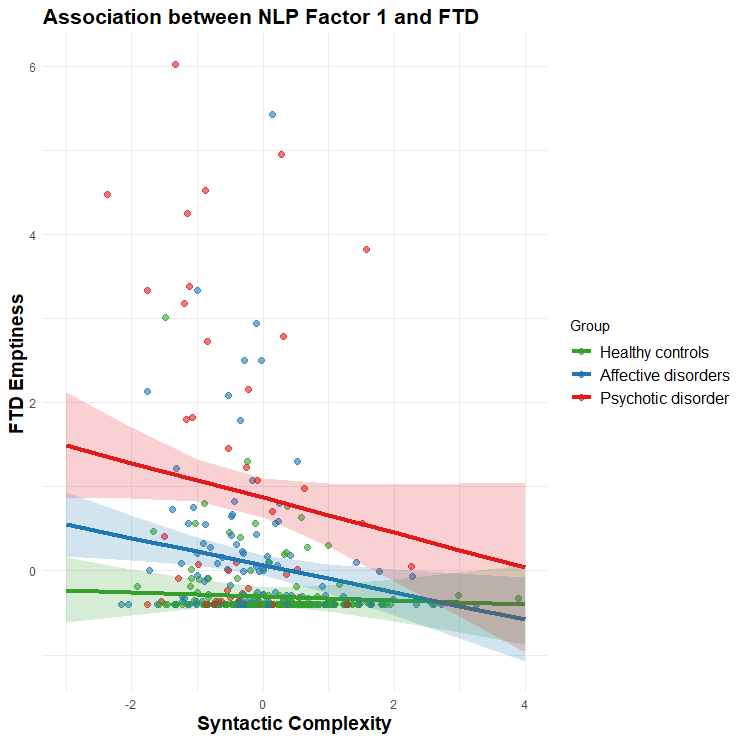
**

1. **Association of** Syntactic Complexity **and FTD Factor Incoherence**

**
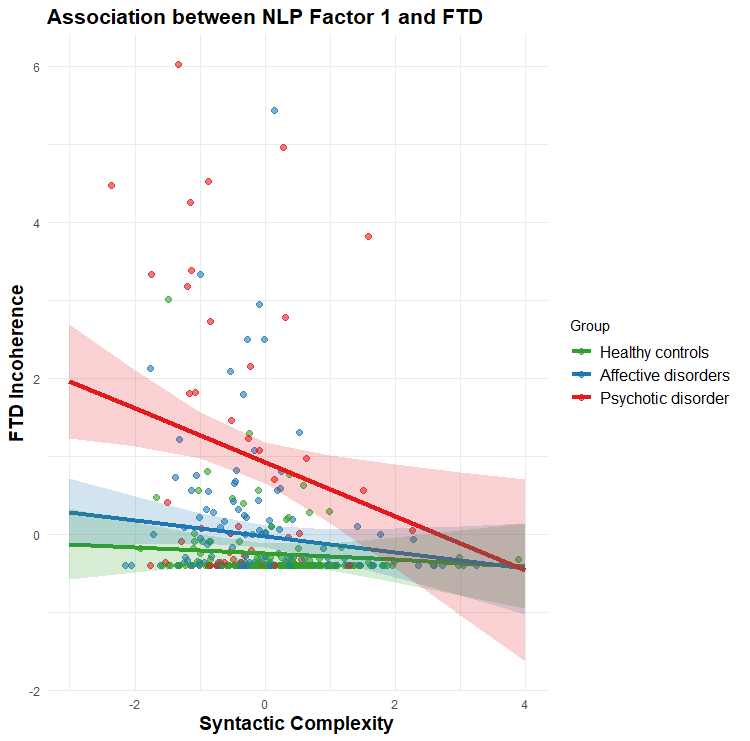
**

1. **Association of** Lexical Diversity and Fluency **and FTD Factor Emptiness**

**
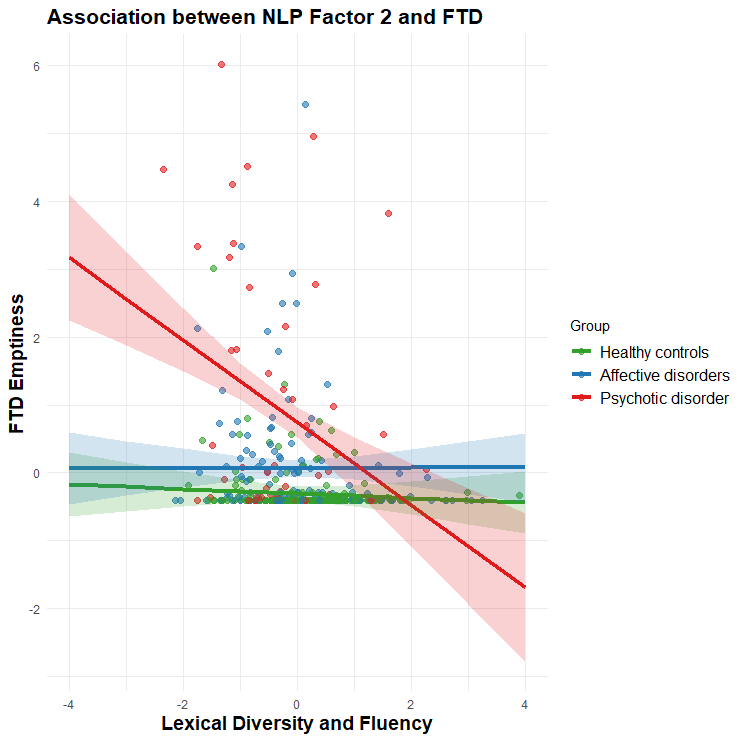
**

### **Supplementary Figure 5.** **Association** **between NLP-Derived Speech Variables and FTD items of the SANS and SAPS scales**


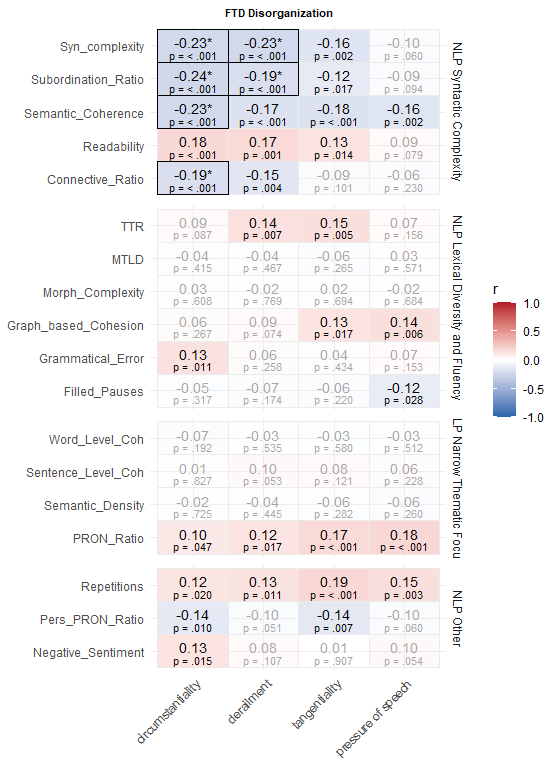


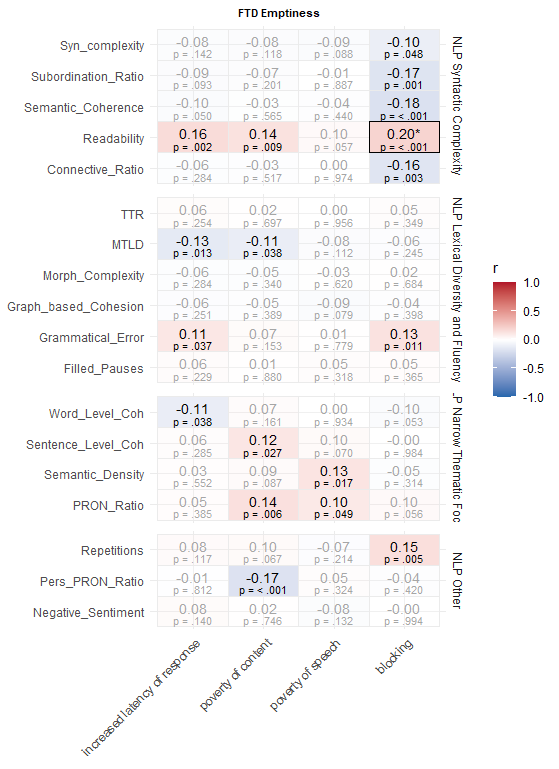


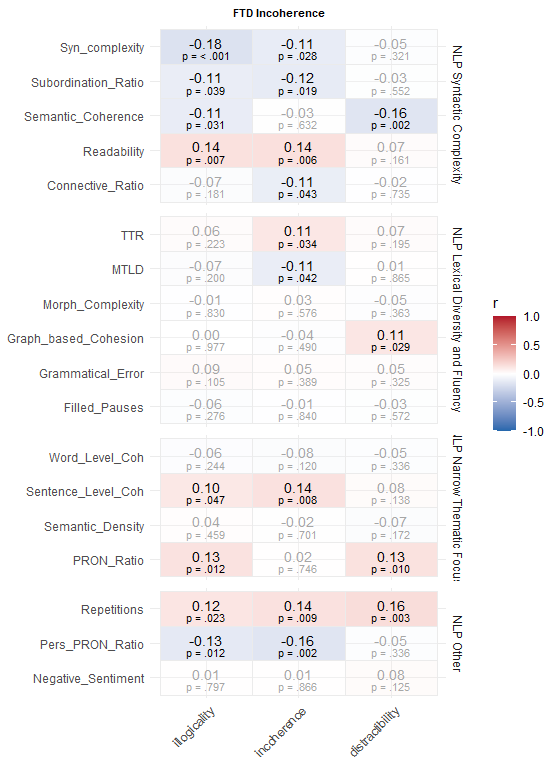


***Notes*. For better readability, non-significant correlations are displayed in grey, while correlations surviving Bonferroni correction are denoted by *.**

## ****eResults 4. Neural Correlates of NLP Speech Factors****

Moderation analyses were performed to test whether IQ or medication load indices (Medication Index, chlorpromazine equivalents, Sackeim Index) influenced the associations between NLP-derived speech factors and neuroimaging findings. For gray matter volume (GMV), these analyses focused on the negative association between Narrow Thematic Focus and GMV in the right posterior insula. None of the moderator variables reached statistical significance (Supplementary Table 5).

For white matter microstructure, moderation analyses assessed whether the observed associations between NLP-derived speech factors and fractional anisotropy (FA) in key tracts were moderated by IQ or medication indices. Across all tracts and speech factors, no moderation effects survived Bonferroni correction for multiple comparisons (Supplementary Table 6).

Additional diffusion tensor imaging (DTI) metrics were examined to further characterize the associations between NLP speech factors and white matter structure. For Syntactic Complexity, lower axial diffusivity (AD) was observed in the right anterior thalamic radiation, left cingulum (cingulate and hippocampal portions), bilateral forceps minor, and left uncinate fasciculus, while higher AD was observed in the right corticospinal tract. Lower mean diffusivity (MD) was also found in the left cingulum (hippocampal portion). For Lexical Diversity and Fluency, higher radial diffusivity (RD) was detected in the right corticospinal tract, left inferior longitudinal fasciculus, right superior longitudinal fasciculus (including the temporal portion), and left uncinate fasciculus, with corresponding higher MD in the left inferior longitudinal fasciculus. For Narrow Thematic Focus, higher RD was observed in the left corticospinal tract, and higher AD in the right inferior longitudinal fasciculus. All results are reported with family-wise error (FWE) correction in Supplementary Table 7.

Finally, the association between formal thought disorder (FTD) dimensions and FA was examined. Negative associations were identified between Disorganization and FA in the right cingulum (hippocampal portion), and between Incoherence and FA in overlapping regions of the right cingulum (hippocampal portion). The detailed results can be found in Supplementary Table 8.

**Importantly, our cohort was not restricted to early-illness cases but consisted predominantly of multi-episode patients, where structural abnormalities are well established and reproducibly observed in large-scale diffusion imaging studies. In this context, the observed associations are best understood as reflecting relatively stable neurobiological substrates of language disturbance rather than transient, treatment-sensitive changes. We believe our current structural findings therefore provide a solid empirical foundation for understanding the neurobiological substrates of computationally derived language features. Overall, the results suggest that NLP-derived speech factors capture structural–linguistic associations with a sensitivity that may exceed that of traditional clinical FTD ratings.**

### ****Supplementary Table 5.**** Moderation Analyses of the Association Between NLP Speech Factor and GMV

| Speech Factor | Brain area | Moderator Variable | ß | SE | 95% CI | *t* | *p* |
| --- | --- | --- | --- | --- | --- | --- | --- |
| *Narrow Thematic* | Right posterior insula | IQ | 0.01 | 0.04 | [-0.08, 0.08] | 0.04 | .966 |
| *Focus* |  | Medication Index | 0.06 | 0.04 | [-0.02, 0.15] | 1.51 | .131 |
|  |  | Chlorpromazin equivalent | 0.09 | 0.05 | [0.00, 0.18] | 1.90 | .058 |
|  |  | Sackeim Index | 0.05 | 0.04 | [-0.02, 0.12] | 1.38 | .168 |

***Note.*  NLP = Natural Language Processing. Moderation analyses were conducted to examine whether IQ or various medication indices moderated the negative association between Speech Factor 3 (**Narrow Thematic Focus**) and GMV in the right posterior insula.**

### ****Supplementary Table 6.**** Moderation Analyses of the Association Between NLP Speech Factors and White Matter Tracts

| Speech Factor | Tract | Moderator Variable | ß | SE | 95% CI | *t* | *p* |
| --- | --- | --- | --- | --- | --- | --- | --- |
| *Syntactic Complexity* | Left ATR | IQ | 0.04 | 0.07 | [-0.09, 0.17] | 0.62 | .535 |
|  |  | Medication Index | -0.09 | 0.07 | [-0.23, 0.04] | 1.38 | .170 |
|  |  | Chlorpromazin equivalent | -0.14 | 0.08 | [-0.30, 0.03] | 1.64 | .103 |
|  |  | Sackeim Index | -0.05 | 0.06 | [-0.17, 0.08] | 0.72 | .469 |
|  | Left UF | IQ | 0.09 | 0.07 | [-0.04, 0.22] | 1.39 | .165 |
|  |  | Medication Index | -0.07 | 0.07 | [-0.20, 0.07] | 0.99 | .323 |
|  |  | Chlorpromazin equivalent | -0.14 | 0.08 | [-0.30, 0.02] | 1.68 | .094 |
|  |  | Sackeim Index | -0.02 | 0.06 | [-0.15, 0.10] | 0.36 | .716 |
| *Lexical Diversity* | Left ATR | IQ | -0.00 | 0.05 | [-0.10, 0.10] | -0.01 | .999 |
| *and Fluency* |  | Medication Index | -0.02 | 0.05 | [-0.12, 0.09] | -0.33 | .739 |
|  |  | Chlorpromazin equivalent | -0.07 | 0.07 | [-0.19, 0.06] | -1.01 | .313 |
|  |  | Sackeim Index | -0.05 | 0.05 | [-0.16, 0.06] | -0.93 | .353 |
|  | Right ATR | IQ | 0.01 | 0.05 | [-0.10, 0.12] | 0.18 | .856 |
|  |  | Medication Index | -0.09 | 0.06 | [-0.20, 0.02] | -1.56 | .120 |
|  |  | Chlorpromazin equivalent | -0.11 | 0.07 | [-0.24, 0.02] | -1.66 | .098 |
|  |  | Sackeim Index | -0.02 | 0.06 | [-0.13, 0.09] | -0.33 | .743 |
|  | Forceps minor | IQ | -0.04 | 0.05 | [-0.14, 0.06] | -0.71 | .480 |
|  |  | Medication Index | -0.05 | 0.05 | [-0.15, 0.06] | -0.88 | .379 |
|  |  | Chlorpromazin equivalent | -0.01 | 0.06 | [-0.14, 0.12] | -0.15 | .884 |
|  |  | Sackeim Index | 0.02 | 0.05 | [-0.09, 0.13] | 0.36 | .722 |
|  | Right ILF | IQ | -0.02 | 0.05 | [-0.11, 0.07] | -0.48 | .632 |
|  |  | Medication Index | -0.01 | 0.05 | [-0.10, 0.09] | -0.18 | .861 |
|  |  | Chlorpromazin equivalent | 0.13 | 0.06 | [ 0.02, 0.24] | 2.24 | .026 |
|  |  | Sackeim Index | -0.06 | 0.05 | [-0.16, 0.03] | -1.26 | .208 |
|  | Right SLF | IQ | 0.04 | 0.05 | [-0.07, 0.14] | 0.68 | .497 |
|  |  | Medication Index | 0.04 | 0.05 | [-0.06, 0.15] | 0.77 | .442 |
|  |  | Chlorpromazin equivalent | 0.09 | 0.07 | [-0.04, 0.22] | 1.42 | .157 |
|  |  | Sackeim Index | 0.01 | 0.05 | [-0.10, 0.12] | 0.14 | .890 |
|  | Left UF | IQ | -0.00 | 0.06 | [-0.11, 0.11] | -0.02 | .982 |
|  |  | Medication Index | 0.02 | 0.06 | [-0.09, 0.13] | 0.33 | .745 |
|  |  | Chlorpromazin equivalent | -0.09 | 0.07 | [-0.22, 0.05] | -1.27 | .207 |
|  |  | Sackeim Index | -0.01 | 0.06 | [-0.13, 0.10] | -0.22 | .828 |
|  | Right UF | IQ | -0.09 | 0.05 | [-0.19, 0.02] | -1.59 | .113 |
|  |  | Medication Index | 0.05 | 0.06 | [-0.06, 0.16] | 0.82 | .413 |
|  |  | Chlorpromazin equivalent | 0.01 | 0.07 | [-0.13, 0.14] | 0.12 | .902 |
|  |  | Sackeim Index | 0.04 | 0.06 | [-0.07, 0.15] | 0.66 | .508 |
| *Narrow Thematic* | Left CST | IQ | -0.04 | 0.06 | [-0.16, 0.08] | -0.66 | .512 |
| *Focus* |  | Medication Index | -0.09 | 0.07 | [-0.21, 0.04] | -1.31 | .192 |
|  |  | Chlorpromazin equivalent | 0.05 | 0.07 | [-0.09, 0.19] | 0.68 | .497 |
|  |  | Sackeim Index | -0.10 | 0.06 | [-0.21, 0.02] | -1.66 | .098 |

***Note.*  NLP = Natural Language Processing. Moderation analyses were conducted to examine whether IQ or various medication indices moderated the negative association between Speech Factors and Fractional Anisotropy. None of the moderation variables reached statistical significance after Bonferroni correction for multiple comparisons across the three NLP-derived speech factors.**

### ****Supplementary Table 7.** Associations Between NLP-Derived Speech Factors and Additional Diffusion Metrics of Axial Diffusivity (AD), Radial Diffusivity (RD), and Mean Diffusivity (MD) Across Key White Matter Tracts**

| Factor | Modality | Correlation | Coordinates of the maximum intensity voxel (x/y/z) MNI | White Matter Tract | Hemisphere | *k* | *P*_FWE_ | β |
| --- | --- | --- | --- | --- | --- | --- | --- | --- |
| ***Syntactic*** | AD | Negative | 21/36/17 | Anterior thalamic radiation | R | 295 | **.009** | -.25 |
| ***Complexity*** |  |  | -15/36/2 | Cingulum cingulate | L | 131 | .023 | -.22 |
|  |  |  | -25/-19/-29 | Cingulum hippocampus | L | 33 | .027 | -.20 |
|  |  |  | 21/36/17 | Forceps minor | L | 623 | **.009** | -.34 |
|  |  |  | -15/36/2 | Forceps minor | L | 553 | **.016** |  |
|  |  |  | -23/38/-3 | Uncinate fasciculus | L | 42 | .034 | -.25 |
|  |  |  | -16/36/-3 | Uncinate fasciculus | L | 16 | .043 |  |
|  | AD | Positive | 20/-11/1 | Corticospinal tract | R | 26 | .045 | .24 |
|  | MD | Negative | -22/-14/-28 | Cingulum hippocampus | L | 42 | .027 | -.19 |
| *Lexical* | RD | Positive | 16/-29/55 | Corticospinal tract | R | 80 | .042 | .30 |
| *Diversity and* |  |  | 12/-27/61 | Corticospinal tract | R | 15 | .047 |  |
| *Fluency* |  |  | 9/-29/66 | Corticospinal tract | R | 13 | .048 |  |
|  |  |  | -38/0/-28 | Inferior longitudinal fasciculus | L | 118 | .031 | .31 |
|  |  |  | -40/3/-31 | Inferior longitudinal fasciculus | L | 11 | .049 |  |
|  |  |  | 41/-4/31 | Superior longitudinal fasciculus | R | 390 | .023 | .30 |
|  |  |  | 55/-10/23 | Superior longitudinal fasciculus | R | 80 | .045 |  |
|  |  |  | 41/-4/31 | Superior longitudinal fasciculus (temporal part) | R | 53 | .035 | .26 |
|  |  |  | -38/0/-28 | Uncinate fasciculus | L | 48 | .041 | .29 |
| *Lexical Diversity and Fluency* | MD | Positive | -45/1/-21 | Inferior longitudinal fasciculus | L | 100 | .043 | .31 |
| *Narrow* | RD | Positive | -24/-21/39 | Corticospinal tract | L | 74 | .036 | .20 |
| *Thematic* |  |  | -23/-28/44 | Corticospinal tract | L | 19 | .046 |  |
| *Focus* | AD | Positive | 34/-58/13 | Inferior longitudinal fasciculus | R | 92 | .033 | .26 |

***Note. N* = 247. R: right, L: left; family-wise-error-corrected p-values. Covariate of no interest included in the regression analyses were age, sex, TIV, and psychiatric diagnoses. Significant results after Bonferroni-correction for the three NLP Speech Factors are in bold.**

### ****Supplementary Table 8.** Negative Association Between Formal Thought Disorder (FTD) Factors and Fractional Anisotropy**

| **Factor** | **Coordinates of the maximum intensity voxel (x/y/z) MNI** | **White Matter Tract** | **Hemisphere** | **k** | **P_FWE_** | **β** |
| --- | --- | --- | --- | --- | --- | --- |
| **Disorganization** | 31/-61/26 | Cingulum/ hippocampus | **R** | 32 | .023 | -.25 |
| **Incoherence** | 22/-53/29 | Cingulum/ hippocampus | **R** | 21 | .043 | -.25 |
|  | 31/-61/26 |  |  | 16 | .036 |  |

***Note.*  N = 242. Due to missing values on the SAPS/SANS items, three observation had to be removed. R: right, L: left; family-wise-error-corrected p-values. Covariate of no interest included in the regression analyses were age, sex, TIV, and psychiatric diagnoses. No significant results after Bonferroni-correction for the three FTD Factors.**

### **Supplementary Note: Subgroup Size and Comparability of the SSD DTI Cohort**

To contextualize the SSD subsample, we examined its demographic and clinical characteristics. The median number of psychotic episodes was 3 (IQR 1–6), with a median age of onset of 17 years, consistent with a multi-episode rather than exclusively first-episode cohort. These features align closely with large-scale DTI studies of chronic or mixed-course schizophrenia, where robust white matter alterations and symptom–brain associations are reproducibly observed [23–25]. Thus, our findings should be interpreted as reflecting established neuropathological processes rather than acute first-episode changes.

Further, the tracts implicated in our analyses—the anterior thalamic radiation (ATR), uncinate fasciculus (UF), and superior longitudinal fasciculus (SLF)—are highly consistent with prior work linking language disturbance and FTD to structural connectivity. For example, de Boer et al. [26] related computational speech metrics to integrity of IFOF, SLF, and UF in schizophrenia, independent of medication effects. Stein et al. [21] demonstrated that FTD dimensions (incoherence, disorganization, emptiness) were associated with ATR and ILF abnormalities across diagnostic groups, without participant overlap with the present study. Schneider et al. [27] reported associations between syntactic complexity and frontotemporal/perisylvian tracts in a smaller sample that partially overlapped with ours.

Taken together, these converging findings suggest that the smaller SSD DTI subgroup does not compromise the validity of our results. Rather, our use of a large transdiagnostic framework, coupled with consistency across independent studies, provides a robust context for interpreting the observed language–brain associations.

## ****Supplementary Analysis S1.** Stability of NLP Factor Structure and Associations with FTD After Excluding Participants with Schizophrenia Spectrum Disorders**

**To evaluate whether the factor structure of NLP-derived speech features and their associations with FTD dimensions were mainly driven by participants with schizophrenia spectrum disorders (SSD), we repeated the exploratory factor analysis (EFA) and correlation analyses with FTD dimensions after excluding all individuals with schizoaffective disorder (*n* = 37) or schizophrenia (*n* = 11). The resulting reduced sample comprised 324 individuals, including healthy controls (*n* = 178) and participants with affective disorders (*n* = 146; Major Depressive Disorder: *n* = 119; Bipolar Disorder: *n* = 27).**

**Exploratory Factor Analysis (EFA)**

**The EFA was conducted using the same analytic pipeline as in the main analyses (unweighted least squares extraction, oblique Promax rotation, and bootstrapping with n = 5000) and yielded an identical three-factor solution. Factor 1 (**Syntactic Complexity**) showed high positive loadings for subordination ratio, connective ratio, syntactic complexity, and semantic coherence, with a strong negative loading for readability. Factor 2 (**Lexical Diversity and Fluency**) loaded strongly on graph-based cohesion, morphological complexity, type–token ratio (TTR), and measure of textual lexical diversity (MTLD), with strong negative loadings for filled pauses and grammatical errors. Factor 3 (**Narrow Thematic Focus**) displayed high positive loadings for pronoun ratio, semantic density, word-level coherence, and sentence-level coherence. The factor structure closely replicated the full-sample solution, with only minor variations in loadings, indicating that SSD participants did not disproportionately influence the dimensionality of speech features. Full bootstrapped loadings are provided in Supplementary Table 9, and the average solution across extraction methods is shown in Supplementary Figure 6.**

**Correlations with FTD Dimensions**

**Bivariate correlations between the NLP factors and the three FTD factors (Disorganization, Emptiness, Incoherence) were recalculated in the reduced sample, with Bonferroni correction for multiple comparisons. Results showed that** Factor 1 (Syntactic Complexity) correlated negatively with FTD Disorganization (*r* = -.21, *p* = .002), as well with FTD Emptiness (*r* = -.18, *p* = .022) and FTD Incoherence (*r* = -.21, *p* = .003). Neither Factor 2 (Lexical Diversity and Fluency) nor Factor 3 (Narrow Thematic Focus) showed significant correlations with FTD factors after Bonferroni correction. T**hese findings indicate that the robust associations between Syntactic Complexity and multiple FTD dimensions generalize beyond SSD, while the Lexical Diversity–Emptiness association appears more specific to patients with psychotic disorders. Importantly, however, the weaker reliability of the Emptiness factor in the reduced sample (Supplementary Table 10) suggests that measurement limitations may also contribute to the absence of a robust association outside the SSD group.**

### **Supplementary Table 9.** Bootstrapped Factor Loadings of Explorative NLP-Derived Speech Factors in the Subsample of Healthy Controls and Patients with Affective Disorders (N = 324)

| Speech Feature | Factor 1 | Factor 2 | Factor 3 |
| --- | --- | --- | --- |
| Subordination ratio | **.818** | .230 | .206 |
| Connective ratio | **.790** | -.025 | .212 |
| Readability | **-.790** | -.211 | .412 |
| Syntactic complexity | **.726** | -.128 | -.054 |
| Semantic coherence | **.700** | -.264 | -.032 |
| Graph-Based Cohesion | -.238 | **.855** | .129 |
| Filled Pauses | .114 | **-.841** | -.278 |
| Morphological complexity | -.200 | **.478** | -.269 |
| TTR | -.255 | **.465** | -.355 |
| MTLD | .020 | **.423** | -.416 |
| Grammatical error | -.265 | **-.458** | -.259 |
| Pronoun ratio | -.278 | .301 | **.767** |
| Semantic Density | -.136 | -.291 | **.638** |
| Word-level coherence | .008 | .207 | **.551** |
| Sentence-level coherence | .270 | -.037 | **.485** |
| Negative sentiment | -.364 | -.088 | .164 |
| Personal pronoun ratio | .087 | .237 | .037 |
| Repetitions | -.070 | -.205 | .027 |

***Note.*  *N* = 324. The extraction method was unweighted least squares with an oblique (promax) rotation. Factor loadings above .40 are in bold. TTR = Type-Token Ratio; MTLD = Measure of Textual Lexical Diversity;** Graph-Based Cohesion **= average shortest path length in a speech graph. Factor 1:** Syntactic Complexity**; Factor 2:** Lexical Diversity and Fluency; Factor 3: Narrow Thematic Focus

### **Supplementary Figure 6. Average Solutions of NLP-Derived Speech Factor Loadings Across Different EFA Methods c F1 = *Syntactic Complexity*, F2= *Lexical Diversity and Fluency*, F3 = *Narrow Thematic Focus*)**


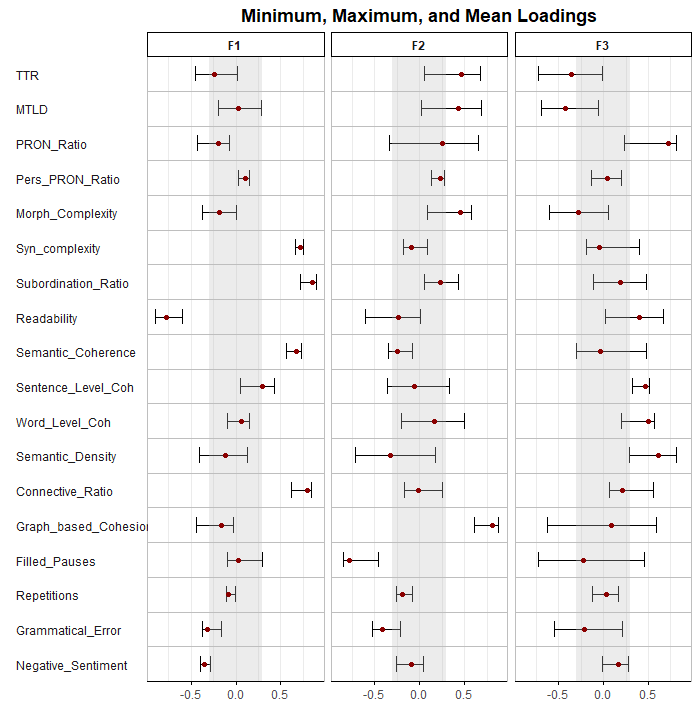


### ****Supplementary Table 10.** Results From a Confirmatory Factor Analysis of Format Thought Disorder (FTD) related SAPS and SANS items in the Subsample of Healthy Controls and Patients with Affective Disorders**

| Factor | Item | Loading | SE | ω |
| --- | --- | --- | --- | --- |
| Disorganization | pressure of speech | **.600** | **.079** | .853 |
|  | circumstantiality | **.548** | **.076** |  |
|  | tangentiality | **.448** | .076 |  |
|  | derailment | **.395** | **.083** |  |
| Emptiness | increased latency of response | **.464** | .130 | .572 |
|  | poverty of content | .282 | .115 |  |
|  | poverty of speech | **.239** | **.089** |  |
|  | blocking | **.131** | **.064** |  |
| Incoherence | illogicality | .301 | .097 | .814 |
|  | incoherence | .178 | .113 |  |
|  | distractibility | .337 | .069 |  |

***Note.*  *N* = 324. The extraction method was unweighted least squares with an oblique (Promax) rotation. Factor loadings above .40 are in bold. ω = Reliability index McDonald's Omega; SANS: Scale for the Assessment of Negative Symptoms; SAPS: Scale for the Assessment of Positive Symptoms**

**References**

**1.** **Explosion. de_dep_news_trf; 2023.**

**2.** **Explosion. spaCy; 2023.**

**3.** **McCarthy PM, Jarvis S. MTLD, vocd-D, and HD-D: a validation study of sophisticated approaches to lexical diversity assessment. Behavior Research Methods. 2010;42:381–92. doi:10.3758/BRM.42.2.381.**

**4.** **Trifu RN, Nemeș B, Bodea-Hațegan C, Cozman D. Linguistic indicators of language in major depressive disorder (MDD). An evidence based research. J Evid-Based Psychot. 2017;17:105–28. doi:10.24193/jebp.2017.1.7.**

**5.** **Hohenheim U. Hohenheimer Verständlichkeitsindex: Fachgebiet für Kommunikationswissenschaft, insbesondere Kommunikationstheorie. 2021. https://komm.uni-hohenheim.de/hohenheimer-verstaendlichkeitsindex. Accessed 21 Oct 2024.**

**6.** **Paul S, Ahrend M-D, Lüers J-C, Roth KS, Grimmiger PP, Bopp F, Janghorban Esfahani B. Systematische Analyse der Lesbarkeit von Patienteninformationstexten auf Internetseiten von Abteilungen für Unfallchirurgie deutscher Universitätskliniken. [Systematic Analysis of Readability of Patient Information on Internet Pages from Departments for Trauma Surgery of German University Hospitals]. Z Orthop Unfall. 2021;159:187–92. doi:10.1055/a-1059-9779.**

**7.** **Bojanowski P, Grave E, Joulin A, Mikolov T. Enriching Word Vectors with Subword Information; 15.07.2016.**

**8.** **deepset. dbmdz German BERT models: Hugging Face; 2019.**

**9.** **Mota NB, Vasconcelos NA, Lemos N, Pieretti AC, Kinouchi O, Cecchi GA, et al. Speech Graphs Provide a Quantitative Measure of Thought Disorder in Psychosis. PLOS ONE. 2012;7:e34928-e34928. doi:10.1371/JOURNAL.PONE.0034928.**

**10.** **Guhr O, Schumann, Anne-Kathrin, Bahrmann F, Böhme HJ. Training a Broad-Coverage German Sentiment Classification Model for Dialog Systems. Proceedings of the Twelfth Language Resources and Evaluation Conference. 2020:1627–32.**

**11.** **Gaser C, Dahnke R, Thompson PM, Kurth F, Luders E, The ADNI. CAT: a computational anatomy toolbox for the analysis of structural MRI data. Gigascience 2024. doi:10.1093/gigascience/giae049.**

**12.** **Jenkinson M, Beckmann CF, Behrens TEJ, Woolrich MW, Smith SM. FSL. NeuroImage. 2012;62:782–90. doi:10.1016/j.neuroimage.2011.09.015.**

**13.** **R Core Team. R: A Language and Environment for Statistical Computing. Vienna, Austria; 2024.**

**14.** **Fabrigar LR, Wegener DT, MacCallum RC, Strahan EJ. Evaluating the use of exploratory factor analysis in psychological research. Psychological Methods. 1999;4:272–99. doi:10.1037/1082-989X.4.3.272.**

**15.** **Sass DA, Schmitt TA. A Comparative Investigation of Rotation Criteria Within Exploratory Factor Analysis. Multivariate Behav Res. 2010;45:73–103. doi:10.1080/00273170903504810.**

**16.** **Braeken J, van Assen MALM. An empirical Kaiser criterion. Psychological Methods. 2017;22:450–66. doi:10.1037/met0000074.**

**17.** **Lorenzo-Seva U, Timmerman ME, and Kiers ,Henk A. L. The Hull Method for Selecting the Number of Common Factors. Multivariate Behav Res. 2011;46:340–64. doi:10.1080/00273171.2011.564527.**

**18.** **Cattell RB. The Scree Test For The Number Of Factors. Multivariate Behav Res. 1966;1:245–76. doi:10.1207/s15327906mbr0102_10.**

**19.** **Steiner M, Grieder S. EFAtools: An R package with fast and flexible implementations of exploratory factor analysis tools. JOSS. 2020;5:2521. doi:10.21105/joss.02521.**

**20.** **Revelle W. psych: Procedures for Psychological, Psychometric, and Personality Research; 2023.**

**21.** **Stein F, Buckenmayer E, Brosch K, Meller T, Schmitt S, Ringwald KG, et al. Dimensions of Formal Thought Disorder and Their Relation to Gray- and White Matter Brain Structure in Affective and Psychotic Disorders. Schizophrenia Bulletin. 2022;48:902–11. doi:10.1093/schbul/sbac002.**

**22.** **Tang S, Hänsel K, Cong Y, Nikzad AH, Mehta A, Cho S, et al. Latent Factors of Language Disturbance and Relationships to Quantitative Speech Features. Schizophrenia Bulletin. 2023;49:S93-S103. doi:10.1093/schbul/sbac145.**

**23.** **Sharkey RJ, Bacon C, Peterson Z, Rootes-Murdy K, Salvador R, Pomarol-Clotet E, et al. Differences in the neural correlates of schizophrenia with positive and negative formal thought disorder in patients with schizophrenia in the ENIGMA dataset. Mol Psychiatry. 2024;29:3086–96. doi:10.1038/s41380-024-02563-z.**

**24.** **Kanaan RA, Kim J-S, Kaufmann WE, Pearlson GD, Barker GJ, McGuire PK. Diffusion Tensor Imaging in Schizophrenia. Biological Psychiatry. 2005;58:921–9. doi:10.1016/j.biopsych.2005.05.015.**

**25.** **Cavelti M, Winkelbeiner S, Federspiel A, Walther S, Stegmayer K, Giezendanner S, et al. Formal thought disorder is related to aberrations in language-related white matter tracts in patients with schizophrenia. Psychiatry Research: Neuroimaging. 2018;279:40–50. doi:10.1016/j.pscychresns.2018.05.011.**

**26.** **de Boer JN, van Hoogdalem M, Mandl RC, Brummelman J, Voppel AE, Begemann MJ, et al. Language in schizophrenia: Relation with diagnosis, symptomatology and white matter tracts. npj Schizophrenia. 2020;6:1–10. doi:10.1038/s41537-020-0099-3.**

**27.** **Schneider K, Alexander N, Jansen A, Nenadić I, Straube B, Teutenberg L, et al. Brain structural associations of syntactic complexity and diversity across schizophrenia spectrum and major depressive disorders, and healthy controls. Schizophrenia (Heidelb). 2024;10:101. doi:10.1038/s41537-024-00517-6.**
